# Identification and characterisation of human apoptosis inducing proteins using cell-based transfection microarrays and expression analysis

**DOI:** 10.1186/1471-2164-7-145

**Published:** 2006-06-12

**Authors:** Ella L Palmer, Andrew D Miller, Tom C Freeman

**Affiliations:** 1 MRC Rosalind Franklin Centre for Genomics Research (RFCGR), Wellcome Trust Genome Campus, Hinxton, Cambridge CB10 1SB, UK; 2 Imperial College Genetic Therapies Centre, Department of Chemistry, Flowers Building, Armstrong Road, Imperial College London, London SW7 2AZ, UK; 3 MRC Laboratory for Molecular Cell Biology and Cell Biology Unit, University College London, Gower Street, London WC1E 6BT, UK

## Abstract

**Background:**

Cell-based microarrays were first described by Ziauddin and Sabatini in 2001 as a powerful new approach for performing high throughput screens of gene function. An important application of cell-based microarrays is in screening for proteins that modulate gene networks. To this end, cells are grown over the surface of arrays of RNAi or expression reagents. Cells growing in the immediate vicinity of the arrayed reagents are transfected and the arrays can then be scanned for cells showing localised changes in function. Here we describe the construction of a large-scale microarray using expression plasmids containing human genes, its use in screening for genes that induce apoptosis when over-expressed and the characterisation of a number of these genes by following the transcriptional response of cell cultures during their induction of apoptosis.

**Results:**

High-density cell-based arrays were successfully fabricated using 1,959 un-tagged open reading frames (ORFs) taken from the Mammalian Gene Collection (MGC) in mammalian expression vectors. The arrays were then used to screen for genes inducing apoptosis in Human Embryonic Kidney (HEK293T) cells. Using this approach, 10 genes were clearly identified and confirmed to induce apoptosis. Some of these genes have previously been linked to apoptosis, others not. The mechanism of action of three of the 10 genes were then characterised further by following the transcriptional events associated with apoptosis induction using expression profiling microarrays. This data demonstrates a clear pro-apoptotic transcriptional response in cells undergoing apoptosis and also suggests the use of common apoptotic pathways regardless of the nature of the over-expressed protein triggering cell death.

**Conclusion:**

This study reports the design and use of the first truly large-scale cell-based microarrays for over-expression studies. Ten genes were confirmed to induce apoptosis, some of which were not previously known to possess this activity. Transcriptome analysis on three of the 10 genes demonstrated their use of similar pathways to invoke apoptosis.

## Background

Cell-based microarray technology was first described by Ziauddin and Sabatini in 2001 [2] for use in performing high throughput over-expression studies. The technique entails printing the full-length ORF of genes inserted into an expression vector onto a glass microscope slide to form a microarray. The array is treated with transfection reagent and cells are grown over the top of the array until confluent. Cells growing in the vicinity of the spots of packaged genes undergo transfection and the encoded protein is over-expressed. Arrays can then be examined for alterations in cellular function, as manifested in localised changes to the cells' biochemistry or morphology. If the expression vector contains a 'tag', the sub-cellular localisation of the protein can also be analysed [2,3]. Due to the techniques' potential for high throughput analyses and economy of reagents, cell-based microarrays for studies have now been adopted by a number of groups for a variety of applications. Cell-based microarray over-expression studies have been used to discover new members of signalling pathways [4], to identify new G-protein coupled receptor (GPCR) targets [5] and to screen single-chain antibody fragments [6]. Comprehensive reviews on cell-based microarrays are available elsewhere [7,8].

Whilst in principle cell-based microarrays provide a powerful platform for performing high throughput transfection screens, to date no studies have reported the use of large-scale arrays and analyses have tended to focus on the over-expression of a relatively small number of genes. One factor that has limited the use of the technology to date has been the availability of suitable clone sets that contain tagged full-length ORF's in mammalian expression vectors, as described in the original paper [2]. Such clone collections are beginning to become available from commercial sources, but for most their use is prohibited by their expense and restrictions on their use. In a previous study [3], we explored the use of GFP tagged genes in Gateway expression vectors in the fabrication of cell-based arrays. Whilst this work demonstrated the utility of using tagged clones in visualising the sub-cellular localisation of the transfected protein, it also highlighted certain limitations with this approach. For example, apart from the considerable expense and time involved in sub-cloning genes into the Gateway cloning system, there is the possibility of introducing errors into the ORF during the initial PCR of the cDNA insert. We also demonstrated that tagging a gene can cause the protein to mis-localise and therefore disrupt the function of the native protein.

In the current study, we have therefore elected to use untagged human cDNA clones from the Mammalian Gene Collection (MGC) [9,10] for the construction of a cell-based microarray capable of screening a large number of genes. There is currently a non-redundant set of over 13,000 sequence verified, full-length ORF human clones in the MGC collection. We have constructed a reverse transfection array containing plasmid DNA from 1,959 of these clones, with each clone printed in quadruplicate. A GFP vector (pEGFP-C1) was also printed to act as a transfection control and to provide a positional address for the untagged MGC clones.

Apoptosis is a mechanism for regulating cell survival by which unwanted or damaged cells are induced to undergo a controlled cell death. During development, apoptosis is used to remove surplus cells and remodel tissues. After birth, apoptosis plays additional roles in tissue homeostasis, immune selection and in deleting cells that have become infected, irreparably damaged, or transformed [11,12]. The apoptotic cascade may be triggered through two major pathways. Extracellular signals such as the tumour necrosis factor (TNF) family of proteins can activate the receptor-mediated extrinsic pathway. Alternatively, stress signals such as DNA damage or withdrawal of survival signals may trigger the mitochondrial intrinsic pathway. Regardless of the mechanism of activation, cells undergoing apoptosis show characteristic features, which include chromatin aggregation, nuclear/cytoplasmic condensation and partitioning of the cytoplasm and nucleus into membrane bound-vesicles (apoptotic bodies), which contain ribosomes, morphologically intact mitochondria and nuclear material [13,14].

Numerous studies have focused on the identification of proteins and pathways that control apoptosis. Apart from the specific interest in understanding how and which proteins regulate the balance between cell survival and cell death, there is considerable interest in regulating or harnessing their activity to modulate disease processes. In the current study, we have employed cell-based microarray technology to screen for genes that are pro-apoptotic when over-expressed. We have then characterised the activity of a number of the genes identified by following the transcriptional response of cell cultures undergoing increased levels of apoptosis due to their over-expression.

In summary, this study has employed a novel approach to the construction of large-scale cell-based microarrays which has been used to screen for proteins that induce apoptosis when over-expressed. Using these arrays we have been able to identify a number of proteins that induce apoptosis, some of which are novel in this respect. Through these studies we have been able to identify common apoptotic pathways on which the different pro-apoptotic proteins appear to be acting.

## Results

**Figure 1:**
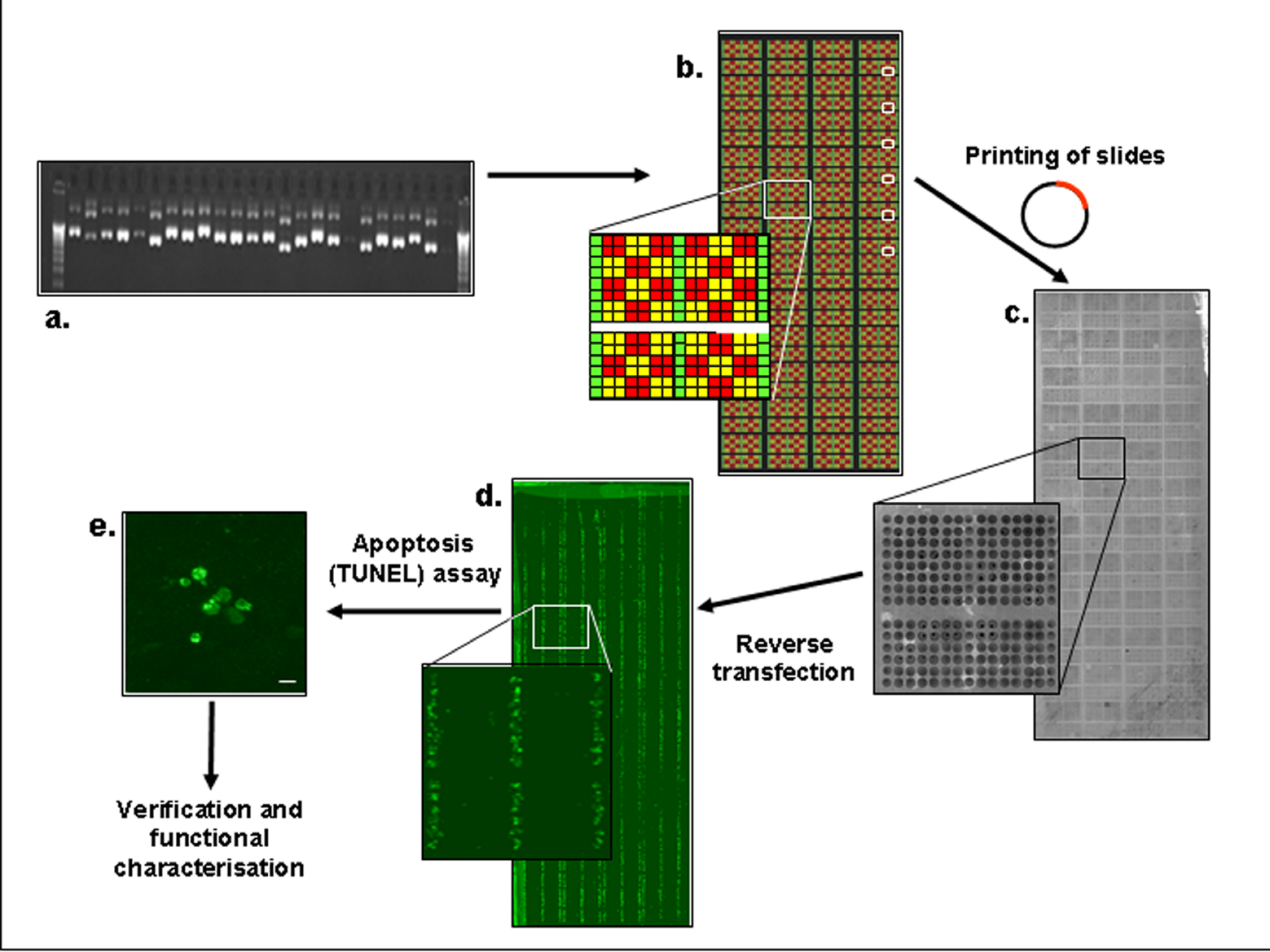
Overview of the design and use of the large-scale cell-based microarray for over-expression studies. a. Representative agarose gel image of plasmids prepared from 2,976 MGC (IRAT) clones. b. Array was designed such that each clone was printed in quadruplicate (yellow and red squares) surrounded by columns of GFP vector (green squares). The position of GFP-tagged positive control genes is shown by small white boxes. c. 1,959 plasmids in 0.3% gelatin were printed on to a glass slide in to form an array with 9,888 features. The image is of an array scanned directly after printing (Agilent microarray scanner). d. An array cultured with HEK293T cells and scanned with a fluorescent imager (GE Healthcare, Typhoon) to show lines of GFP positive cells. e. Arrays were subjected to a functional assay to detect changes in the cell after over-expression of proteins. The image is of TUNEL positive cells, scale bar = 10 μm.

Microarrays were constructed using plasmid DNA from 1,959 human MGC clones in the pCMV-SPORT6 vector. All plasmids were printed in quadruplicate within grids (Figure 1b) except GFP, which was printed in columns to demarcate the grids and to act as a control for transfection. Seven genes in the Gateway pcDNA-DEST47 C-terminal GFP fusion vector for which we have previously characterised sub-cellular localisations [7] were also included as transfection controls. The arrays were incubated with HEK293T cells for approximately 40 hours, after which time clear grids of the transfection control pEGFP-C1 could be observed (Figure 1d). Four separate arrays on two separate occasions were subjected to the Terminal Deoxynucleotide Transferase dUTP Nick End Label (TUNEL) assay, which allows the detection of free 3'-OH termini present in the fragmented DNA of apoptotic cells (e). Replication of assays was found to be crucial for stringent screening as the ability to obtain a result for an individual gene was often compromised by imperfections in the cell monolayer, either as a result of differences in cell growth or post-culture treatment of the slides. A protein was scored as positive for apoptosis if one TUNEL-positive cell was observed by eye over one or more of the four replicate spots. This was a deliberately low threshold for a positive readout. The small number of cells over each spot (30–50), and the fact that apoptotic cells could be cleared in the late stages of apoptosis meant that the signal for even a strong pro-apoptotic gene was potentially weak. 79 of the 1,959 genes (4%) represented on the array were scored positive in two of the four replicate assays and were therefore considered to be potentially pro-apoptotic.

The 79 genes were then scrutinised further by examining their activity when transfected in 6-well plates. The results from the 6-well experiment indicated that out of the 79 positives from the array, 69 (87.3%) showed no higher rates of cell death in cultures following transfection than the controls, with 10 (12.7%) being true positives. The function, GO descriptions and localisations were obtained for the 10 pro-apoptotic proteins identified by this study (Table 1). Some of these are known to be involved in apoptotic pathways, while others are uncharacterised in this respect (see Discussion for a full description of these genes and their potential mechanism of action).

Gene Ontology (GO) categorisation lists 11 genes on the array under the apoptosis category. Only one of these genes (STK3) was observed in the final list of ten positive genes. Assuming that all 11 of the GO categorised genes are capable of inducing apoptosis when over-expressed, the false negative rate of the array based assay can be estimated as 91%. From these numbers the sensitivity could be estimated as 60%, with an assay specificity measured by the positive predictive value of 12.1% (see Methods for details of calculations).

**Figure 2:**
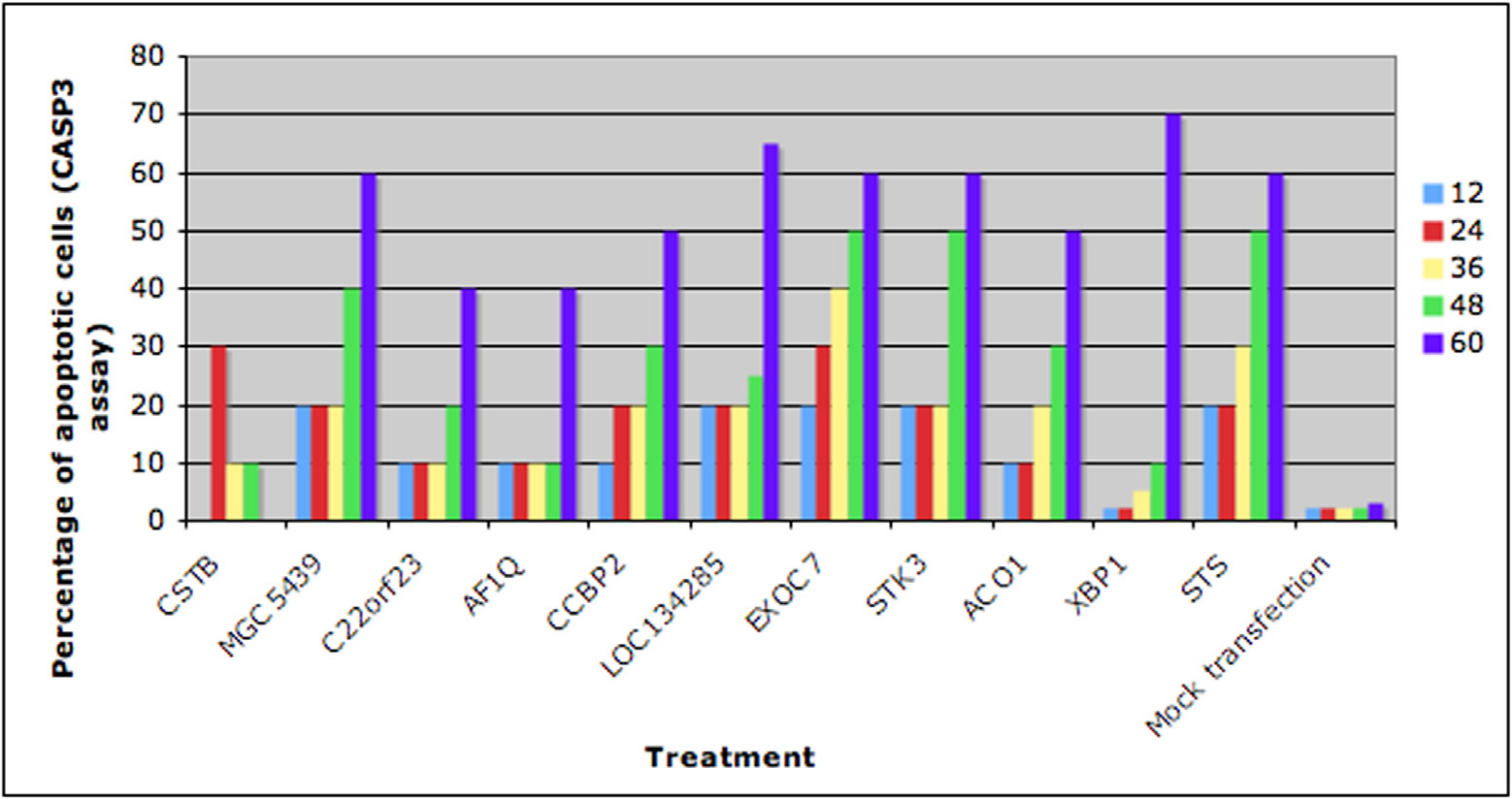
Percentage of apoptotic cells after over-expression of the 10 pro-apototic genes. STS treatment and mock transfection in 6 well plate CASP3 assays.

To determine the dynamics of apoptosis induction brought about by the over-expression of the 10 pro-apoptotic proteins, all were analysed in plate-based assays, using both the TUNEL and cleaved-CASP3 assays at 12, 24, 36, 48 and 60 hours following transfection. A mock transfection and a well-characterised inducer of apoptosis, staurosporine (STS) were assayed in parallel at each time point, as negative and positive controls respectively. Higher levels of apoptosis were observed in cell cultures over-expressing each of the 10 proteins and the positive (STS) control relative to the negative control, with levels of apoptosis rising at later time points (Figure 2).

**Figure 3:**
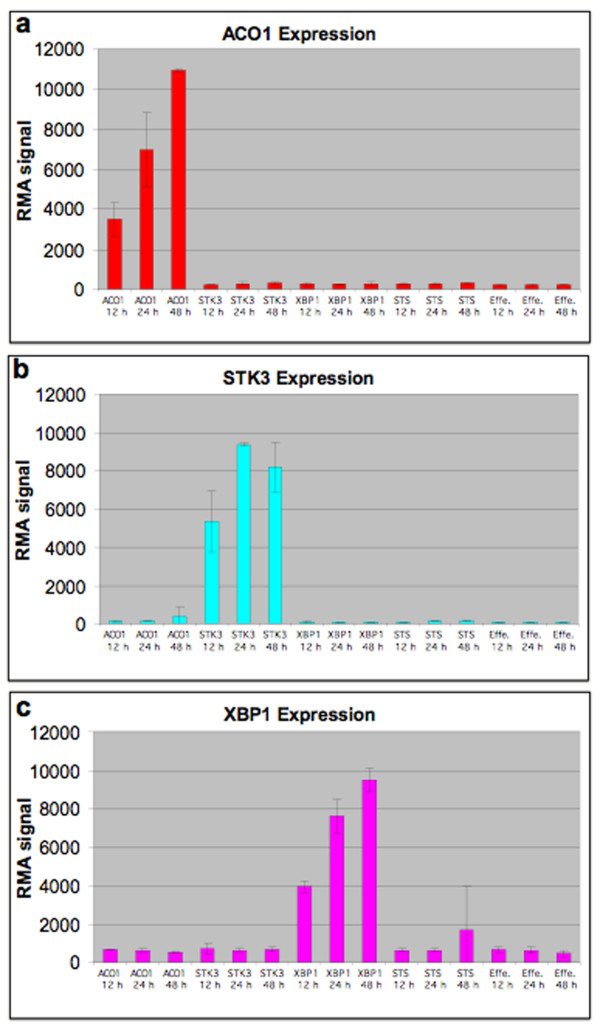
*ACO1*, *STK3 *and *XBP1 *expression following transfection in six-well plates. *ACO1*, *STK3 *and *XBP1 *probe set RMA normalised signal intensities were plotted over the time course for each combined sample. Results for replicate samples were averaged, error bars indicate the individual replicate measurements.

Three of the 10 proteins, ACO1, STK3 and XBP1 were chosen for further characterisation, by expression profiling of cell cultures over-expressing the genes. Following transfection, 12, 24 and 48 hour time points were chosen from the time-course study in order to cover early, mid and late gene transcriptional events associated with apoptosis progression (). RNA samples were labelled and hybridised to the Affymetrix HG-U133 GeneChip and the data was normalised using both the GeneChip Operating System (GCOS) and Robust Multivariate Analysis (RMA). All raw and normalised data has been submitted to ArrayExpress [1] (Accession Number: E-MEXP-421). The data was of high quality with the chips showing little variation in the quality control parameters recorded in the GCOS report file. Box plots also showed data distributions to be relatively similar across all chips (Additional file 1 Figure One). Non-supervised clustering of the data using the conditions tree function within GeneSpring (Agilent Technologies) suggested that there was little or no treatment or time-specific clustering of the data (Additional file 2 Figure Two). *ACO1*, *STK3 *and *XBP1 *appeared to be constitutively expressed within the cells as assessed by the GCOS software, with these genes being reported as being present (P) in all the samples at all the time points (data not shown). *XBP1 *appeared to have the highest constitutive expression i.e. gave the greatest signal, followed by *ACO1*, then *STK3*. The signal intensities for all three transcripts (*ACO1*, *STK3 *and *XBP1*) increased up to 41, 70 and 12 times, respectively, within cells where the genes were over-expressed compared with the average expression level in cell cultures in which the genes had not been transfected. Mock transfected and STS treated cells showed no rise in any of the three gene transcripts above constitutive levels. Despite apparent differences in the baseline level of expression of the three gene transcripts, all genes showed similar expression levels (signal) following transfection (Figure 3). These three genes exhibited the most significant fold changes in their expression following transfection relative to any of the genes represented on the Affymetrix array.

**Figure 4:**
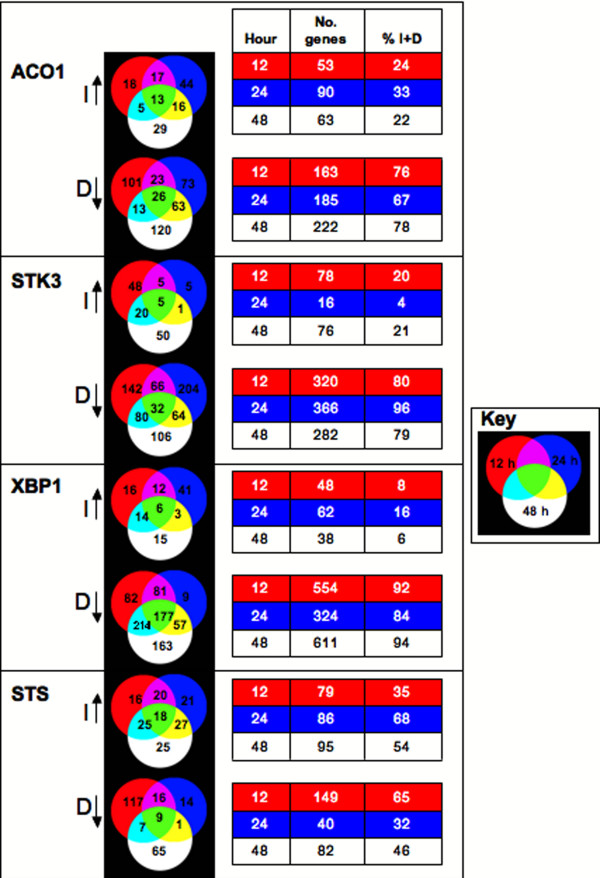
Venn diagrams prepared with the list of 997 differentially expressed transcripts. I↑ and ↓D, number of genes increased or decreased in expression respectively, compared to the mock transfection control at that time point. Venn diagrams, 12 hours (red), 24 hours (blue), 48 hours (white). Boxes; No. genes – gene number increasing or decreasing at that time point, % I+D – percentage of genes increasing or decreasing at that time point.

**Figure 5:**
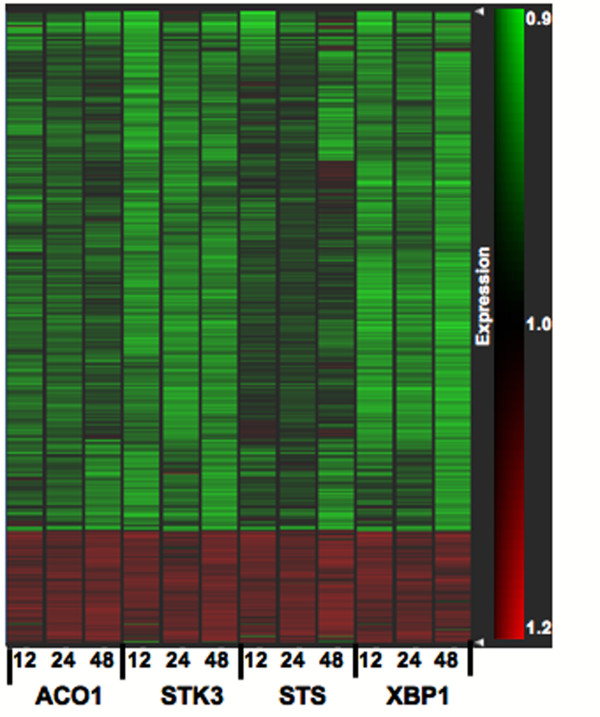
Gene tree prepared with the list of 997 differentially expressed transcripts. The tree was generated using the Spearman correlation algorithm within GeneSpring. Expression is shown in fold change compared to the appropriate mock transfection control. Red – genes increased in expression; Green – genes decreased in expression; Black – unchanged.

Data normalised using the RMA method, was then used to prepare a list of differentially expressed genes. ANOVA analysis was used to compare replicate data from each of the four test conditions (three over-expressed genes plus STS treatment) at each time point with the appropriate negative (mock transfected) control data. Overall, 3,791 gene transcripts were observed to be significantly differentially expressed in at least one of the 12 pair-wise comparisons. To refine the list of differentially expressed transcripts and minimise the false discovery rate, only transcripts that showed a fold change greater than 1.4 and appeared in three or more of the 12 individual pair-wise comparisons were analysed further. This 'Differentials List' (Additional file 3 Table One) contained 997 transcripts. Based on this list, Venn diagrams were prepared from the individual comparisons such that the overlap in genes either differentially under or over-expressed across time-points could be assessed (Figure 4). Overall, there was considerable overlap in the genes found to be differentially expressed across time-points, but there also appeared to be many that were specific to individual time points. Of all the transcripts found to be differentially expressed following transfection, an average of 78.2% across all time points and transfections were observed to decrease in expression. However, the overall number of gene transcripts that increased and decreased in expression was similar following treatment with STS. There were more transcripts in the *XBP1 *differentials lists than with other treatments and there were no obvious time point-specific trends in the number of gene transcripts increasing or decreasing in expression across the conditions. Supervised clustering of the 997 transcripts present in three or more of the individual comparisons was used to assess the behaviour of these genes across the four experimental conditions. This revealed a surprising uniformity in the behaviour of genes across different time points and treatments (Figure 5). Whilst individual genes showed some variation across the data set, genes that were up or down regulated in one comparison tended to show the same behaviour across all comparisons, although this change didn't always reach the level of statistical significance.

In an effort to associate the genes found to be differentially expressed in this experiment with apoptosis, we utilised the University of Michigan list of apoptosis regulators [15]. This resource was used as it listed a large number of genes that according to the literature are potentially involved in apoptotic pathways. Of the 1,099 apoptosis regulators listed on this site, 130 were present in the differentials list generated by this experiment. This 'Apoptosis Differentials List' was therefore a list of differentially expressed genes that have been previously associated with apoptosis (Additional file 4 Table Two). The Apoptosis Differentials List contained genes that belonged to the same family and there were also genes that had known interaction partners (see Discussion and Additional file 4 Table Three for more detail).

In an effort to piece this information together, proteins within the Apoptosis Differentials List were marked red if they are thought to be pro-apoptotic and green if they appeared to increase the likelihood of cell survival based on the literature (Additional file 4 Table Two). If genes could be categorised in this way, we endeavoured to place them on a simplified Kyoto Encyclopedia of Genes and Genomes (KEGG) apoptosis pathway to give an indication of their likely site of activity within the apoptotic pathway.

**Figure 6:**
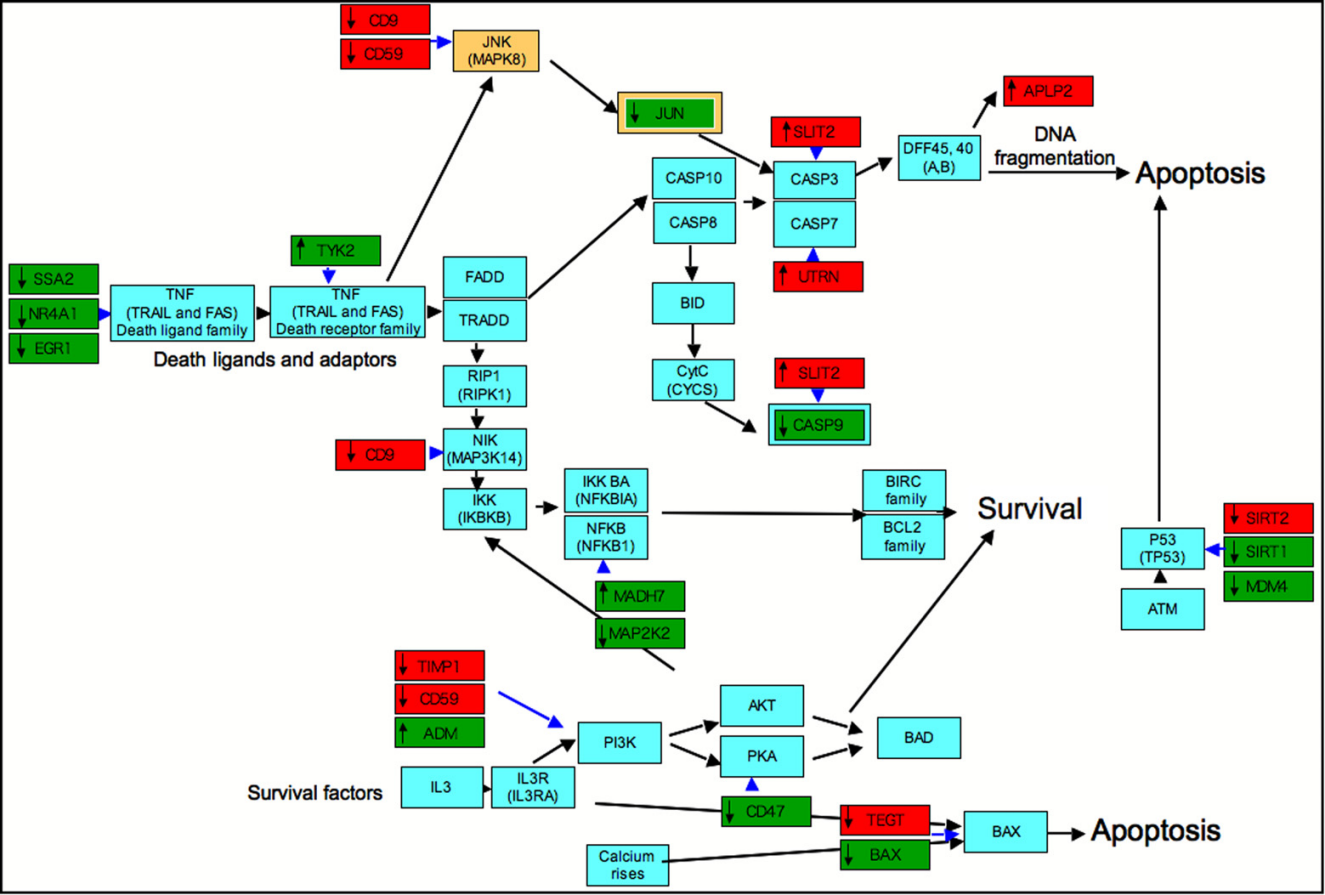
Summary of the effects of gene over-expression on the mRNA levels for genes associated with apoptotic pathways. Central pathway has been adapted from the KEGG apoptotic pathways (light blue) and BD Biosciences apoptotic pathway (light orange). A gene is shown on the pathway if present in at least one of the time points in at least three of the 4 individual treatments. Arrows within the gene mRNA boxes indicate genes with increased or decreased expression. Genes coloured red potentially increase apoptosis, genes coloured green potentially decrease apoptosis according to the literature (see Additional file 4 Table Two and Three and Additional file 5 Figures Three to Seven for Apoptosis Differentials List and more comprehensive pathways).

Figure 6 attempts to summarise this information by showing genes/pathways that appear to be central to apoptosis induction in the current study (see Additional file 5 Figures Three to Seven for separate figures of the simplified KEGG pathway for each time point/transfected gene).

## Discussion

The aim of this study was to adopt a functional genomics approach to screen for human genes that induce apoptosis when over-expressed. In order to do this, our strategy required the construction of a microarray of a large number of human genes in mammalian expression vectors. In a previous study, GFP-tagged genes in Gateway expression vectors were used to examine the sub-cellular localisation of proteins over-expressed by reverse transfection [3]. In common with other studies, we found that gene-tagging can disrupt the normal sub-cellular localisation and therefore presumably the function of the protein. In addition, sub-cloning of the gene inserts has the potential to introduce errors in the ORF during vector construction and inserting large numbers of genes into Gateway constructs is both costly and time consuming. Therefore, for the present study we decided to adopt a new approach to the construction of a high-content microarray of human genes by direct use of the readily available full-length MGC clones [9,10]. This strategy was possible as many of the MGC clones were already in the pCMV-SPORT6 vector, which contains a CMV promoter to drive expression of the ORF in mammalian cells. Plasmid preparations were attempted for 2,976 MGC clones but we found that many did not yield enough/any product, despite repeated attempts. However, more than 2 μg plasmid DNA was recovered from 1,959 clones. In order to maximise the likelihood of observing the effects of over-expression on the cells covering arrays without compromising the array content, each of the 1,959 purified MGC clones was printed in quadruplicate onto a glass slide to form the array. After inclusion of control features, the array possessed 9,888 features in total. As such, this represents the largest over-expression cell-based reverse transfection microarray published to date.

Following growth of HEK293T cells over the array, the TUNEL assay was used to detect genes which had induced cell death when over-expressed. The assay was repeated on four separate arrays. When proteins only positive in two or more of the four assays were taken into account, 79 of the 1,959 genes (4%) appeared to be potentially inducing cell death. For verification, these 79 genes were then transfected in 6-well plates. The results from the 6-well plate assay indicated that out of the 79 positives from the array, 10 (12.7%) were true positives (Table 1). This would indicate that the arrays gave a fairly high false positive rate. Calculation of the false negative rate presents more of a challenge. However, in an attempt to evaluate this we determined the number of genes on the array that have been linked with apoptosis by Gene Ontology (GO) categorisation. Eleven genes on the array were listed under the apoptosis category, but only one of these genes (STK3) was observed in the final list of 10 positive genes. Assuming that all of these genes are capable of inducing apoptosis when over-expressed, the false negative rate of the array based assay was estimated as 91%. Whether all 11 genes are truly capable of inducing apoptosis if over-expressed and whether they would do so in all cell types is uncertain. The high false positive and estimated false negative rates of this assay are attributable to a number of factors. In order to maintain the positional address of the positive signals relative to the marker gene (GFP) transfections during manual inspection of the arrays, it was necessary to view the array at a relatively low magnification (×10 objective). At this resolution the relatively weak signal produced by TUNEL assay may have been overlooked or background signal attributed incorrectly. Employing automated high resolution scanning and image analysis tools would not only make scoring of cell-based arrays a lot easier, but would also improve the accuracy of scoring. The necessity for spot recognition software and the storage and analysis of the images present a considerable challenge. These issues are currently being explored and a microscope-based screening platform is being developed with automated sample preparation, image acquisition and data analysis [16,17]. Improved tools for automated image analysis of cell-based arrays are also being developed elsewhere [18].

Of the 10 proteins found by this study to induce apoptosis when over-expressed a number have been linked previously with apoptosis, others have not. *STK3 *is known to be involved in Fas-mediated apoptosis and is cleaved/activated by CASP3 [19]. In addition, it is possible to hypothesise mechanisms of action of four of the other proteins identified. XBP1 binds to the X-box of the HLA-DR-alpha promoter (MHC human class II gene) [20] and responds to accumulation of unfolded proteins in the ER [21]. It is possible that this change may trigger apoptosis, although this has not been demonstrated previously. There is other evidence that XBP1 may be linked to apoptosis. In an expression profiling study of murine mammary epithelial cells expressing conditionally active STAT3 (which provides an essential death signal for mammary epithelial cells following weaning), *XBP1 *was highly up-regulated following STAT3 activation [22]. CSTB maintains appropriate equilibrium between free cysteine proteases and their complexes [23]. Cathepsins can be inhibited by CSTB and therefore this could prevent degradation of peptides and proteins, which in turn could possibly act as a pro-apoptotic stimulus. ACO1 represses ferritin and increases TFR translation [24], and its over-expression is therefore likely to cause a build up of free-iron within the cell. Previous studies have shown that increased levels of intracellular free-iron can induce apoptosis [25]. EXOC7 is a component of the exocyst complex involved in the docking of exocystic vesicles with fusion sites on the plasma membrane [26] and potentially excessive removal of internal cell contents may cause apoptosis to occur. MLLT11 and CCBP2 are both found in tumours, but no other information is available to allow speculation on a possible mode of action for their induction of apoptosis. The same is true of the relatively uncharacterised genes C22ORF23, MGC5439 and LOC134285.

From even the limited number of 'pro-apoptotic' genes identified by this study, it would appear that they could be further classified into two categories; those that are 'true' apoptotic modulators, the proteins they encode being directly involved with the cell's apoptotic/survival machinery; and those that act indirectly, their over-expression leading to cytotoxic changes within the cell which then triggers a pro-apoptotic response. It is inevitable that screens such as this will expose genes in both categories and further work is necessary to determine their exact mode of action. Whilst 'true' apoptotic modulators might be considered more interesting in the pursuit of improved understanding of the apoptotic cascade, genes that give rise to apoptosis by indirect mechanisms may still have interest as novel therapeutic agents, for example in cancer gene therapy.

In an attempt to ascertain the timing and strength of apoptosis, a time course transfection study using HEK293T cells was undertaken on all 10 genes, a positive control STS and a mock transfected negative control at 12, 24, 36, 48 and 60 hours following transfection. Staurosporine (STS) was used as the positive control as it is an apoptotic effector classically linked to caspase activation [27]. As the TUNEL assay has been shown to potentially detect necrotic death in addition to apoptotic death [28], a cleaved CASP3 assay was performed on the cultures in addition to the TUNEL assay. STS and all 10 genes identified by the reverse transfection screen led to cultures in 6-well plates exhibiting between 40–70% TUNEL and cleaved CASP3 positive cells after 60 hours compared with only 3–4% in the mock transfection cultures.

Apoptosis has been studied extensively and core pathways and events are generally well established. The regulation of apoptotic cell death is a complex interplay between proteins that promote cell survival and those that promote cell death. It is widely thought that the processes that control the balance between the life and death of a cell are regulated exclusively at the post-transcriptional level. As a result few observations have been made of the transcriptome during this process, although those that have [29] suggested that this would be a useful approach to further characterise the action of these genes.

*STK3*, *ACO1 *and *XBP1 *were selected over the other seven genes for expression profiling studies (Figure 2) as the proteins they encode had either been shown in previous studies to induce or be connected to apoptosis (*STK3 *and *XBP1*) or a clear apoptotic hypothesis could be postulated (*ACO1*). For the expression profiling study, samples were taken at 12, 24 and 48 hours following transfection to observe the early, mid and late transcriptional events associated with apoptosis. *ACO1*, *STK3 *and *XBP1 *were all found to be constitutively expressed within the cells but at different levels. It was interesting to note that despite differences in constitutive expression, all genes appeared to reach a similar level of up-regulation following transfection and that the changes in expression observed for these transcripts were the largest of all the transcripts represented on the expression profiling array.

It was envisaged that expression profiling experiments might reveal a transcriptional response in these cultures that would provide clues as to the mechanism by which the over-expression of these genes induces apoptosis. Overall, analysis of the microarray expression data indicated that the changes observed at all time points and across all conditions were relatively subtle. There was no strong tendency for the data to cluster according to treatment or time-point (Additional file 2 Figure Two). The lists of differentially expressed transcripts of genes prepared by comparing each time point with its respective mock transfection control, showed many genes to be significantly changing their expression, but on the whole these changes were relatively small i.e. the majority of changes were less than 2-fold in magnitude. For each transfection experiment, many more transcripts were down-regulated in expression than up-regulated during apoptosis progression. A similar observation was also reported in a previous study of the transcriptional events associated with apoptosis following removal of cell survival factors from cultures of HUVEC cells [29]. However, with STS treatment the number of gene transcripts that were significantly down-regulated and up-regulated in expression overall was similar. What this indicates with respect to the fundamental mode of action for STS in apoptosis induction as opposed to the gene over-expression is uncertain. The number of transcripts identified as significantly changing with each treatment and at each time point varied considerably. In order to compare the condition or time-specific changes in transcript expression between the genes, a gene tree was plotted of the 997 transcripts that had changed in expression more than 1.4-fold and that were present in 3 or more of the 12 pair-wise treatment comparisons (Figure 5). There was a surprising degree of uniformity in behaviour of these genes across all conditions and time points. Genes that were up- or down-regulated in one comparison tended to show the same behaviour across all comparisons, although this change did not always reach the level of statistical significance. Indeed, we were unable to find any convincing condition-specific changes in transcriptome activity that might give clues as to the mechanism by which functionally distinct genes induce apoptosis when over-expressed. Whether this is because the events leading to the initiation of a pro-apoptotic response occurred post-transcriptionally or that the changes are too subtle to be recognised is unclear. Rather, these findings strongly indicate that the majority of the changes we observed were associated with a universal pattern of gene regulation during apoptosis, regardless of the initiating trigger.

In order to further explore the apoptotic signatures in this data, the University of Michigan list of apoptosis regulators [15] was used to identify other potentially interesting apoptosis-associated genes in the list of differentials. 130 genes were shared between this list and the list of genes found here to be differentially expressed. Many gene family members and known interactors that have been previously associated with either the pro-apoptotic or cell survival machinery were present within this new list of 130 apoptosis-associated genes. (The full list of these genes and an in depth discussion of their potential role in apoptosis induction or cell survival is available in the Additional file 4 Tables Two and Three). A literature search was performed on the 130 genes in the list to ascertain their function. Some apoptosis associated genes e.g. *NR4A1*, *EGR1 SLIT2*, *CASP9*, *ADM*, *SMAD7*, *JUN *and *TIMP1 *significantly changed in their expression in every transfection/treatment compared to the negative control; others were only observed to change under certain conditions. In order to provide a simplified view of this data, these genes were mapped onto a modified version of the KEGG apoptosis pathway if they were present in at least three of the four experimental conditions. Their action in either increasing or decreasing the likelihood of apoptosis and their directional change in expression is indicated (Figure 6, also see Additional file 5 Figures Three to Seven for apoptosis pathways and further discussion for each of the over-expressed gene and STS treatments). Overall, this approach supported the hypothesis that the transfected genes and STS were ultimately acting through similar pathways to induce cell death. In each case, numerous genes were observed to change which were associated with the MAPK8(JNK)/CASP3 pathway and in addition there were clear indications of suppression of the cell survival pathways. Whilst the KEGG pathway was helpful in visualising the apoptotic pathways, many of the genes on the University of Michigan list of apoptosis regulators [15] had to be added onto the pathway. In addition, there are most likely other genes in the lists of differentials that will be influencing the progression of apoptosis, but have not yet been recognised as being involved with the regulation of cell death.

Overall, the expression profiling studies have provided valuable insights into the transcription changes associated with apoptosis, as the transcriptional changes associated with programmed cell death have not been studied extensively. This current study supports the notion that there are discrete changes in the mRNA abundance of certain genes during apoptosis [29]. As many of these transcripts encode proteins that are known regulators of cell survival and death, it would seem likely that transcriptional regulation of these mediators contributes to a cell's final decision to undergo a programmed cell death.

## Conclusion

This study reports the design and use of the first truly large-scale cell-based microarray for studies using the approach described first by Ziauddin and Sabatini [2]. It has demonstrated the potential as well as the current limitations of this technology to screen large numbers of genes for those that induce a functional change in cellular physiology when over-expressed. We have identified 10 genes by this methodology that can induce apoptosis when transfected into HEK293 cells, some of which have been previously associated with apoptosis and others which have not. In order to examine the functional activity of these genes, a time course expression profiling experiment was set up to follow the transcriptional changes associated with apoptosis induction for three of the genes. This revealed that apoptosis induction is associated with discrete changes in a cell's transcriptome and that many of these changes seem identical regardless of mechanism by which apoptosis has been induced. Furthermore, many of the genes observed to change in their expression level during cell death have previously been associated with apoptosis and in this study strongly indicate the activation of the MAPK8(JNK)/CASP3 and BCL2 pathways.

## Methods

### Clone purification

MGC clones [8,9] in IRAT plates 1–21 and 36–45 were purchased from MRC geneservice [30]. Replicate working plates were prepared by adding 1 μl of IRAT plate clones to 2X TY media containing 8% glycerol (Sigma, Gillingham, Dorset, UK) and 50 μg/ml ampicillin (Sigma), grown at 37°C overnight and stored at -20°C. Clones from each IRAT plate were grown and purified four times. Clones were grown by adding 10 μl of the working plate clones to 1 ml 2X TY media containing 50 μg/ml ampicillin and grown for 26 h at 37°C in a shaking incubator at 320 rpm. Clones were purified as described in the MultiScreen_96 _PLASMID plate kit protocol (Millipore, Watford, UK).

pEGFP-C1 (Clontech, Cowley, Oxford, UK) was propagated as described in the DH5α *E. coli *kit protocol, (Invitrogen, Paisley, UK), glycerol stocks were prepared from 850 μl of the culture and 150 μl of glycerol and stored at -70°C. 10 μl of pEGFP-C1 glycerol stock was added to 5 ml 2X TY media with 100 μg/ml kanamycin (Sigma) and 10 μl glycerol stocks of CXADR, MARKL1, TGIF, CDK9, NFIB, IL17BR, TNFRSF10B Gateway C-terminal GFP destination vector pcDNA-DEST47 (Invitrogen) prepared as described previously [7] were added to 5 ml 2X TY media with 100 μg/ml ampicillin and grown in a shaking incubator at 37°C for 8 h. The pEGFP-C1 was transferred to flasks containing 100 ml 2X TY media with 100 μg/ml kanamycin and pcDNA-DEST47 and pCMV-SPORT6 clones were transferred to flasks containing 100 ml 2X TY media with 100 μg/ml ampicillin and grown at 37°C for 16 h in a shaking incubator, the clones were purified as described in the plasmid midiprep kit protocol (Qiagen, Crawley, West Sussex, UK).

IRAT clones for the 10 apoptotic inducing genes; XBP1, CSTB, MGC5439, STK3, C22ORF23, ACO1, MLLT11, CCBP2, LOC134285 and EXOC7 were streaked out on 2X TY agar plates containing 100 μg/ml ampicillin and incubated overnight at 37°C. A single colony was picked from each. XBP1, CSTB, MGC5439, STK3 and C22ORF23 were grown up and purified as the pcDNA-DEST47 clones described above. ACO1, MLLT11, CCBP2, LOC134285 and EXOC7 were grown up in 5 ml 2X TY media with 100 μg/ml ampicillin at 37°C for 12–16 h with vigorous shaking and plasmids were prepared as described in the Wizard miniprep kit protocol (Promega, Southampton, UK). The 96-well Picogreen dsDNA Quantitation Kit (Molecular probes, Paisley, UK) and the Cytofluor 4000 with Cytofluor software (Applied Biosystems, Warrington, UK) and Cytocalc (Applied Biosystems) were used to quantify 96-well miniprep purified IRAT clones. Concentrations were adjusted to a 200 ng/ml control. A Jenway Spectrophotometer (Genova Lifescience, UK) was used to measure all other clone purifications. All samples were electrophoresed on 1% ethidium bromide agarose gels.

### Re-array plates

Plasmid DNA from IRAT plates 1–21 and 36–45 with concentrations over 2 μg were re-arrayed into 21 fresh 96-well flat bottomed plates. The DNA was dried via a heated vacuum centrifuge (Eppendorf, Westbury, US), H_2_O was added back to a 0.5 μg/μl concentration and the plates were stored at -20°C.

### Printing of cell-based transfection arrays

1 μg IRAT plasmids, pEGFP-C1 vector (Clontech) and TNFRSF10B, IL17BR, NFIB, CDKN1B, NFIL3 and PTPN11 in the pcDNA-DEST47 vector (Invitrogen) were made up to 30 μl with 0.3% gelatin (Sigma) and transferred into 384-well plates. Clones were printed onto poly-lysine slides (Sigma) using a Biorobotics MicroGrid II Microarrayer (Biorobotics, Cambridge, UK) with a 48 pin head (Quill pins 2500, Biorobotics). The microarrayer was programmed to print 16 single pre-spots, 12 spots with a 25 ms dwell with two 7 sec, 60°C wash and dries between picking up clones. To determine if the DNA had printed correctly, arrays were scanned with Cy3 and Cy5 lasers on a DNA microarrayer scanner (Agilent Technologies, West Lothian, UK), the resultant .tif files were split using Tiff splitter A5.1.1.1 (Agilent Technologies) and viewed with the software program Image Analysis A.5.1.1 (Agilent Technologies). Each spot was ~140 μm in diameter. Arrays were stored desiccated at 4°C.

### Transfection of arrays

16 μl Enhancer (Effectene transfection reagent kit, Qiagen) and 150 μl EC buffer (Qiagen) per array were incubated at RT for 5 min, 25 μl Effectene (Qiagen) was added and the total volume pipetted onto the array. Parafilm (Teklab, Durham, UK) was cut to the size of the slide was lowered onto the array and the transfection reagent incubated on the array at RT for 20 min. The parafilm and transfection reagent was removed, and arrays were placed in a 10 × 10 cm square dish (BD biosciences, Cowley, Oxford, UK). Human embryonic kidney (HEK293T) cells were grown and maintained in 500 ml DMEM with 0.11 g/l NA PYR with pyroxidine containing 50 ml FCS, 100 U/ml penicillin, 100 μg/ml streptomycin (Invitrogen) at 37°C and 5% CO_2_. 1 × 10^7 ^HEK293T cells were incubated at 37°C, 5% CO_2 _for 24 h before reverse transfection, after 24 h, 1 × 10^7 ^cells in a total of 20 ml culture medium were carefully poured into the 10 × 10 cm dish and incubated at 37°C, 5% CO_2 _for 40 h.

### 6-well plate transfections

22 mm coverslips (VWR) were coated with 0.01% poly-l-lysine solution (Sigma) and placed in 6-well plates. The 79 positive clones from the reverse transfection screen were added in Effectene transfection reagent (Qiagen) according to manufacturer's recommendations to 2 × 10^5 ^HEK293T cells and incubated for 40 h. Cover slips were removed and the TUNEL assay performed (see below). For the time course, all cell cultures were set up at the same time and transfections or STS treatment were performed at different points during culture, so all cell cultures were at the same growth phase when harvested. Cells were seeded into 5 wells per treatment/time point, then the 10 verified positive apoptotic IRAT clones, a negative control (transfection reagent only) and a positive control (final concentration 1 μm staurosporine (STS)) were added to the cells for 12, 24, 36, 48 and 60 h, then the TUNEL and cleaved CASP3 assay applied (see below). Cells were removed and frozen from three wells per treatment/condition for the time course and stored at -70°C for subsequent Affymetrix microarray sample preparation.

### TUNEL and cleaved CASP3 assays

For the TUNEL assay, cells were fixed with 1% paraformaldehyde (38% paraformaldehyde (VWR, Dorset, UK), diluted with PBS) for 10 min and the protocol followed as described in the Apoptag Apoptosis Detection System kit protocol (Flowgen, Nottingham, UK). 200 μl TdT enzyme/reaction buffer, anti-digoxigenin antibody/blocking solution and blocking solution were added per cell-based microarray and 50 μl per 6-well plate coverslip.

For the cleaved CASP3 assay, cells were fixed with 3.8% paraformaldehyde for 20 min and the protocol followed as described for the cleaved caspase-3 (Asp175) antibody with fluorescein conjugate (Cell Signalling Technology, Beverly, Massachusetts, USA). 50 μl of diluted cleaved CASP3 antibody was added per 6-well plate coverslip, a circle of parafilm (Teklab, Durham, UK) was applied to ensure even coverage of antibody and incubated shielded from light at 4°C for 8 h.

### Cell visualisation and counting positive cell fluorescence

A drop of mounting medium containing DAPI stain (Vector, Peterborough, UK) was applied to a glass slide coverslip (Agilent) for the cell-based microarrays or to the 6-well plate coverslip and lowered onto the cell-based microarray or a standard glass microscope slide for the 6-well plate coverslips (Amersham Biosciences, Buckinghamshire, UK). Fluorescence was primarily visualised using a Typhoon scanner (Amersham Biosciences) with a resolution of 50 μm to determine if transfection had occurred. This level of resolution was not high enough to analyse the transfection events on the arrays, therefore an Eclipse E800 microscope (Nikon, Kingston Upon Thames, UK) with a confocal attachment (BioRad, Hemel Hempstead, UK) was used to analyse the arrays at ×10 magnification. At this magnification, the GFP positive controls could be used as a positional tool. Positives were recorded as a quadruplicate clone patch with one or more fluorescent cells. Each microarray was scored twice and the genes were ranked in Excel according to the number of positives. For the TUNEL and cleaved CASP3 6-well plate assays, cells were scored as positive if clear fluorescent apoptotic bodies were observed in or near the cells. The number of cells in patches of cleared cells were estimated and included in the total percentage of apoptotic cells. Slides were stored at 4°C.

### Statistical analysis of transfection assays

The distribution of probabilities was calculated based on constant probability.

The number of measurable array positions for each plasmid was divided by the probability distribution and the number of positive's expected by chance for each plasmid was calculated. This number was compared to the actual number observed on the arrays.

To calculate the sensitivity of the arrays, the equation True Positive (TP)/(True Positive (TP) + False Negative (FN)) was used. TP was calculated as the number of positives in the 6-well assay follow up experiments. FN was calculated as the number of known tyrosine apoptosis inducing genes (determined using Gene Ontology) on the reverse transfection array that should have been positive, but were not found to be positive.

The equation True Positive TP/True Positive (TP)+False Positive (FP) was used to calculate the Positive Predictive Value of the arrays. False Positive (FP) was calculated as the number of genes that were found to be positive in half of more of the reverse transfection arrays, but in the follow up 6-well plate assay were not found to be positive.

### Sample preparation for Affymetrix GeneChip microarrays

RNA was extracted as described in the RNeasy mini kit protocol (Qiagen) from the three frozen time course samples for *STK3*, *ACO1*, *XBP1 *transfections and the positive and negative control. cDNA was prepared using the T7-(dT)_24 _primer (5'ggccagtgaattgtaatacgactcatagggaggcgg-(dT)_24_3') (SigmaGenosys) as described in the SuperScript DoubleStranded cDNA synthesis kit (Invitrogen) from the two samples per condition with the highest RNA yields. cDNA was purified as described in the 1.5 ml Heavy Phase lock Gel (PLG) tubes protocol (Fisher Scientific, Loughborough, UK). Biotin labelled cRNA was prepared by *in vitro *transcription (IVT) from the cDNA as described in the Bioarray High Yield RNA transcript labelling kit (Enzo, New York, USA) and cleaned up as described in the RNeasy Mini kit (Qiagen). The cRNA was fragmented as described in the GeneChip Expression Analysis Affymetrix Manual [31]. RNA, cRNA and fragmented cRNA was checked on the Agilent 2100 Bioanalyzer using the RNA 6000 assay (Agilent, West Lothian, UK) with universal RNA as a control (Stratagene, Cambridge, UK) and RNA 6000 ladder (Ambion, Huntingdon, UK). cDNA and RNA was quantified with the Nanodrop (Labtech International Ltd, East Sussex, UK). The hybridisation cocktail was prepared and the GeneChips hybridised and washed as the GeneChip eukaryotic control kit (Affymetrix) and the GeneChip Expression Analysis Affymetrix Manual [31]. The GeneChips were scanned using the Scanner 3000 (Affymetrix) and the GeneChip Operating System (GCOS) (Affymetrix).

### Data analysis

The .cel data was normalised using GCOS (Affymetrix) and Robust Multichip Analysis (RMA), which is available as a programme, called 'affy' from Bioconductor [32]. The .txt GCOS and RMA normalised data were loaded into GeneSpring 7 (Silicon Genetics, Redwood City, California). A list of genes differentially expressed between each condition time point and the negative control sample at the relevant time point was prepared in 3 stages: 1. the GCOS normalised data was filtered to remove any gene not flagged as being present (P) in both replicate samples in at least one condition/time point. 2. The GCOS Present lists (1.) were used to filter the RMA normalised data. The cross gene error model (CGEM) and a parametric analysis of variance between groups (ANOVA) statistical test was applied and the false discovery rate was set at 0.05. 3. ANOVA lists (2.) were further filtered to remove any gene whose change was less than 1.4 fold were removed. The Differentials gene lists were entered into Excel and a pivot table of genes was prepared and ordered according to the number of times the genes appeared over the whole condition set (see Additional file 3 Table One for lists of that appear in at least three conditions). Differentially regulated genes present in more than two conditions were visualised using Venn diagrams for each condition at each time point. The HUGO approved names were obtained for the University of Michigan list of apoptosis regulators [15], combined with the lists of differentially changed genes and a list of apoptotic genes was obtained (see Additional file 4 Table Two for full apoptosis list). Genes from the apoptosis list with an apoptotic pathway recorded from the literature were mapped onto a simplified Kyoto Encyclopedia of Genes and Genomes (KEGG)/BD Biosciences pathway [33,34].

## Authors' contributions

EP carried out all the experimental work and participated in the design of the study. TF and AM conceived of the study and participated in its design.

## Supplementary Material

Additional File 1 Box plots showing data before and after RMA normalisation. a) before b) after. The box of a boxplot demonstrates the 25^th ^to 75^th ^percentile of the data and the line within the box indicates the median. The length of the box represents the difference between the 25^th ^and 75^th ^percentiles and the lines either side of the box indicate the largest and smallest values which are not outliers. Click here for file

Additional File 2 Condition tree of all time course samples. The tree was generated with RMA normalised data with the Spearman correlation algorithm within GeneSpring. Red ACO1, Yellow Effectene, Pink XBP1, Blue STS, Light blue STK3. Click here for file

Additional File 3 Differentials List. Table 1. RMA normalised data was used to prepare a list of differentially expressed genes by comparing replicate data using an ANOVA analysis from each of the four test conditions (three over-expressed genes plus STS treatment) at each time point with the appropriate negative mock transfected control. Overall, 3,791 gene transcripts were observed to be significantly differentially expressed in at least one of the 12 comparisons. To minimise the false discovery rate only the 997 gene transcripts that showed a fold change greater than 1.4 and appeared in more than two of the 12 individual comparisons were analysed further. Click here for file

Additional File 4 Table 2 Apoptosis Differentials List/Table 3 Families and gene interactors in Apoptosis Differentials List. (Table 2) Genes from Differentials Lists that were present in the University of Michigan list of apoptosis regulators and apoptosis GO ontologies (GO genes indicated in light yellow if additional to the Univeristy of Michigan list). Genes were ranked depending on the number of times they occurred in the 12 time course samples compared to the negative mock transfection control at that time point. Affymetrix probe sets for the same gene were grouped together. Dark blue = genes decreased in expression by 2-fold or more. Light blue = genes decreased in expression between 1.4 and 2 fold. Dark pink = genes increased in expression 2 fold or over. Light pink = genes increased in expression between 1.4 and 2 fold. White = no change in expression compared to the negative control. APOP red = when over expressed, genes increase apoptosis according to the literature. APOP green = when over expressed, genes decrease apoptosis according to the literature. APOP white = no confirmation via literature whether an increase or decrease in apoptosis is caused by the gene change in expression. EXPT = consequent action in this experiment dependent on whether gene expression is increased or decreased. Red = increases apoptosis. Green = decreases apoptosis. White = No confirmation via literature of apoptotic effect therefore unable to deduce role in this experiment. If genes only occurred in one sample at one time point, they were only included if the fold change compared to the appropriate mock transfection control was more than 1.6. (Table 3) Families and gene interactors in Apoptosis Differentials List (some genes are included from the Differentials List). Click here for file

Additional File 5 Figure 3. ACO1 over-expression effect on apoptotic pathway. Figure 4. STK3 over-expression effect on apoptotic pathway. Figure 5. XBP1 over-expression effect on apoptotic pathway. Figure 6. STS over-expression effect on apoptotic pathway. Figure 7. Summary of over-expression effects in apoptotic pathways. (Figures 3, 4, 5, 6, 7) Adapted from the KEGG apoptotic pathway (light blue) and BD Biosciences apoptotic pathway (light orange). Genes coloured red potentially increase apoptosis, genes coloured green potentially decrease apoptosis dependent upon their expression (red and green genes may be increased or decreased in expression, see Table 2. White writing indicates the changed expression in the gene was only observed in ACO1. Click here for file

## Figures and Tables

**Table 1:** Summary of the 10 genes found in these studies to induce apoptosis when over-expressed. Function, sub-cellular localisations and GO categories of the genes are given. Yellow highlights GO apoptotic activity.

**Gene name**	**Function**	**Proposed apoptotic hypothesis**	**Localisation (Swiss-Prot)**	**GO categories**
**C22ORF23**	Not known	None	Not known	Not known

**XBP1**	XBP1 binds to the X box of the HLA-DR-alpha promoter (MHC human class II gene) [20] and responds to accumulation of unfolded proteins in the ER [21].	XBP1 increases ER membrane production, this change in intra-cellular balance may trigger apopotosis.	Nuclear	- Immune response - Nucleus - Regulation of transcription - Transcription factor activity

**CSTB**	The main role of cathepsins is the degradation of protein. Maintenance of appropriate equilibrium between free cysteine proteases and their complexes with inhibitors is crucial for proper functioning of all living systems [23]. Mutations in CSTB result in myoclonus epilepsy [35].	If cathepsins are highly inhibited by CSTB, this could prevent degradation of peptides and proteins, which could stimulate an apoptotic pathway.	Cytoplasmic and nuclear.	- Cysteine protease inhibitor - Nucleus

**MGC5439**	Not known	None	Not known	Not known

**STK3**	STK3 and 4 are involved in Fas mediated apoptosis and are cleaved/activated by CASP3. When stably expressed in HeLa cells, STK3 and 4 highly sensitise the cell to death receptor mediated apoptosis by accelerating CASP3 activation [19].	These findings suggest that STK3 and STK4 play a role in apoptosis both upstream and downstream of caspase activation [16].	Cytoplasmic (By similarity)	- Protein kinase CK2 activity - Serine/threonine kinase activity - cAMP-dependent kinase activity - Cytoplasm - Smino acid phosphorylation - Tyrosine kinase activity - Transferase activity - **Apoptosis** - ATP binding - Signal transduction

**ACO1**	Transferrin receptor (Tfr) is a membrane receptor that transports iron into the cell via endocytosis. Free iron is toxic to cells, but not if bound to ferritin. ACO1 (IREBP – IRE binding protein) [25] represses ferritin translation and increases Tfr translation [24].	Over-expression of ACO-1 will cause increased Tfr and excess iron within the cell. Ferritin translation is repressed by ACO1 so the excess free iron will be unbound. Excess intracellular iron induces apoptosis in cells.	Cytoplasmic	- Lyase activity - Tricarboxylic acid cycle - Aconitate hydratase activity - Cytoplasm - Negative regulation of translation - Metabolism - RNA binding

**MLLT11**	Involved in acute leukemias by a chromosomal translocation. MMLT11 could possibly be a cytokine [36].	None	Not known	- Cell growth and/or maintenance

**CCBP2**	CCBP2 is a promiscuous receptor [37], binds to SCYA2/MCP-1, SCY3/MIP-1-ALPHA, SCYA5/RANTES AND SCYA7/MCP-3. CCBP2 is expressed in the lymphatic endothelium, a subset of vascular tumors [37] and melanoma cells [38].	None	Integral membrane protein	- Immune response - Integral to plasma membrane - Development - G-protein coupled receptor - Chemotaxis - C-C chemokine receptor activity - Rhodopsin-like receptor activity

**LOC134285**	No information			-Receptor activity

**EXOC7 (KIAA1067)**	EXOC7 is 1 of 8 subunits of the exocyst which transports material within membrane bound vesicles inside the cell to the surface. The intracellular vesicles fuse with the plasma membrane and contents are released to the exterior [26].	Over-expression of EXOC7 may cause excessive removal of internal cell contents and cause apoptosis to occur.	Cytosolic	- Intracellular protein transport - Exocytosis - Exocyst - Protein transporter activity
