# Supplementary material for: Identification and characterisation of human apoptosis inducing proteins using cell-based transfection microarrays and expression analysis
Source: BMC Genomics. 2006 Jun 12;7:145. doi: 10.1186/1471-2164-7-145 (PMC1525185; doi:10.1186/1471-2164-7-145)
Supplement: Additional File 1 — Box plots showing data before and after RMA normalisation. a) before b) after. The box of a boxplot demonstrates the 25th to 75th percentile of the data and the line within the box indicates the median. The length of the box represents the difference between the 25th and 75th percentiles and the lines either side of the box indicate the largest and smallest values which are not outliers. [file 1471-2164-7-145-S1.doc]

Figure 1. Box plots showing data before and after RMA normalisation. a) before b) after. The box of a boxplot demonstrates the 25^th^ to 75^th^ percentile of the data and the line within the box indicates the median. The length of the box represents the difference between the 25^th^ and 75^th^ percentiles and the lines either side of the box indicate the largest and smallest values which are not outliers. Effectene labels the mock-transfected cells.
