# Supplementary material for: Identification and characterisation of human apoptosis inducing proteins using cell-based transfection microarrays and expression analysis
Source: BMC Genomics. 2006 Jun 12;7:145. doi: 10.1186/1471-2164-7-145 (PMC1525185; doi:10.1186/1471-2164-7-145)
Supplement: Additional File 2 — Condition tree of all time course samples. The tree was generated with RMA normalised data with the Spearman correlation algorithm within GeneSpring. Red ACO1, Yellow Effectene, Pink XBP1, Blue STS, Light blue STK3. [file 1471-2164-7-145-S2.doc]

Figure 2. Condition tree of all time course samples. The tree was generated with RMA normalised data with the Spearman correlation algorithm within GeneSpring. Red ACO1, Yellow Effectene, Pink XBP1, Blue STS, Light blue STK3. Effectene labels the mock-transfected cells.
