# Supplementary material for: Identification and characterisation of human apoptosis inducing proteins using cell-based transfection microarrays and expression analysis
Source: BMC Genomics. 2006 Jun 12;7:145. doi: 10.1186/1471-2164-7-145 (PMC1525185; doi:10.1186/1471-2164-7-145)
Supplement: Additional File 3 — Differentials List. Table 1. RMA normalised data was used to prepare a list of differentially expressed genes by comparing replicate data using an ANOVA analysis from each of the four test conditions (three over-expressed genes plus STS treatment) at each time point with the appropriate negative mock transfected control. Overall, 3,791 gene transcripts were observed to be significantly differentially expressed in at least one of the 12 comparisons. To minimise the false discovery rate only the 997 gene transcripts that showed a fold change greater than 1.4 and appeared in more than two of the 12 individual comparisons were analysed further. [file 1471-2164-7-145-S3.doc]

**Table 1. Differentials List. RMA normalised data was used to prepare a list of differentially expressed genes by comparing replicate data using an ANOVA analysis from each of the four test conditions (three over-expressed genes plus STS treatment) at each time point with the appropriate negative mock transfected control. Overall, 3,791 gene transcripts were observed to be significantly differentially expressed in at least one of the 12 comparisons. To minimise the false discovery rate only the 997 gene transcripts that showed a fold change greater than 1.4 and appeared in three or more of the 12 individual comparisons were analysed further (shown here).**
