# Supplementary material for: Identification and characterisation of human apoptosis inducing proteins using cell-based transfection microarrays and expression analysis
Source: BMC Genomics. 2006 Jun 12;7:145. doi: 10.1186/1471-2164-7-145 (PMC1525185; doi:10.1186/1471-2164-7-145)
Supplement: Additional File 4 — Table 2 Apoptosis Differentials List/Table 3 Families and gene interactors in Apoptosis Differentials List. (Table 2) Genes from Differentials Lists that were present in the University of Michigan list of apoptosis regulators and apoptosis GO ontologies (GO genes indicated in light yellow if additional to the Univeristy of Michigan list). Genes were ranked depending on the number of times they occurred in the 12 time course samples compared to the negative mock transfection control at that time point. Affymetrix probe sets for the same gene were grouped together. Dark blue = genes decreased in expression by 2-fold or more. Light blue = genes decreased in expression between 1.4 and 2 fold. Dark pink = genes increased in expression 2 fold or over. Light pink = genes increased in expression between 1.4 and 2 fold. White = no change in expression compared to the negative control. APOP red = when over expressed, genes increase apoptosis according to the literature. APOP green = when over expressed, genes decrease apoptosis according to the literature. APOP white = no confirmation via literature whether an increase or decrease in apoptosis is caused by the gene change in expression. EXPT = consequent action in this experiment dependent on whether gene expression is increased or decreased. Red = increases apoptosis. Green = decreases apoptosis. White = No confirmation via literature of apoptotic effect therefore unable to deduce role in this experiment. If genes only occurred in one sample at one time point, they were only included if the fold change compared to the appropriate mock transfection control was more than 1.6. (Table 3) Families and gene interactors in Apoptosis Differentials List (some genes are included from the Differentials List). [file 1471-2164-7-145-S4.doc]

**Table 2. Apoptosis Differentials List. Genes from Differentials Lists that were present in the University of Michigan list of apoptosis regulators and apoptosis GO ontologies (GO genes indicated in light yellow if additional to the Univeristy of Michigan list). Genes were ranked depending on the number of times they occurred in the 12 time course samples compared to the negative mock transfection control at that time point. Affymetrix probe sets for the same gene were grouped together. Dark blue = genes decreased in expression by 2-fold or more. Light blue = genes decreased in expression between 1.4 and 2 fold. Dark pink = genes increased in expression 2 fold or over. Light pink = genes increased in expression between 1.4 and 2 fold. White = no change in expression compared to the negative control. APOP red = when over expressed, genes increase apoptosis according to the literature. APOP green = when over expressed, genes decrease apoptosis according to the literature. APOP white = no confirmation via literature whether an increase or decrease in apoptosis is caused by the gene change in expression. EXPT red = consequent increase in apoptosis in this experiment according to the literature. EXPT green = consequent decrease in apoptosis in this experiment according to the literature. EXPT white = no confirmation via literature of apoptotic effect therefore unable to deduce role in this experiment. If genes only occurred in one sample at one time point, they were only included if the fold change compared to the appropriate mock transfection control was more than 1.6.**

| **NO.** | **GENE NAME** | **PPROBE** | **A**  **12** | **A**  **24** | **A**  **48C4** | **SK12** | **SK24** | **SK48** | **XB12** | **XB24** | **XB48** | **SS12** | **SS24** | **SS48** | **FUNCTION AND REFERENCE** | **APOP** | **EXPT** |
| --- | --- | --- | --- | --- | --- | --- | --- | --- | --- | --- | --- | --- | --- | --- | --- | --- | --- |
| 1 | NR4A1 | 202340_x_at |  |  |  |  |  |  |  |  |  |  |  |  | NR4A1 induces FASL and TRAIL (Rajp*al et* al, 2003). |  |  |
| 2 | CASP9 | 203984_s_at |  |  |  |  |  |  |  |  |  |  |  |  | CASP9 binds to APAF1 (Takata*ni et* al, 2004). |  |  |
| 3 | SNAI2 | 213139_at |  |  |  |  |  |  |  |  |  |  |  |  | SNAI2 resists withdrawal of survival factors/pro-apoptotic signals cell death (Ve*ga et* al, 2004). |  |  |
| 4 | FZD7 | 203706_s_at |  |  |  |  |  |  |  |  |  |  |  |  | FZD encode WNT proteins which act through TCF, JNK and calcium pathways (Kirikoshi and Katoh, 2002, Herin and Sheng, 2002). |  |  |
|  |  | 203705_s_at |  |  |  |  |  |  |  |  |  |  |  |  |  |  |  |
| 5 | TIMP1 | 201666_at |  |  |  |  |  |  |  |  |  |  |  |  | TIMP1 inhibits apoptosis, mediated through PI3K and AKT (Vorotniko*va et* al, 2004, L*ee et* al, 2003). |  |  |
| 6 | BAX | 211833_s_at |  |  |  |  |  |  |  |  |  |  |  |  | BAX binds and antagonises BCL2 and causes apoptosis via CASP3 (Willis, et al., 2003). |  |  |
|  |  | 208478_s_at |  |  |  |  |  |  |  |  |  |  |  |  |  |  |  |
| 7 | SLIT2 | 209897_s_at |  |  |  |  |  |  |  |  |  |  |  |  | SLIT2 may activate apoptotic pathways through CASP3 and CASP9 (Dall*ol et* al, 2003). |  |  |
|  |  | 230130_at |  |  |  |  |  |  |  |  |  |  |  |  |  |  |  |
|  |  | 228850_s_at |  |  |  |  |  |  |  |  |  |  |  |  |  |  |  |
| 8 | MDM4 | 235589_s_at |  |  |  |  |  |  |  |  |  |  |  |  | Increased MDM4 enhances stabilistaion of stress-induced p53 and promotes apoptosis (Manci*ni et* al, 2004). |  |  |
|  |  | 236814_at |  |  |  |  |  |  |  |  |  |  |  |  |  |  |  |
| 9 | EGR1 | 201694_s_at |  |  |  |  |  |  |  |  |  |  |  |  | Gene targets mediiated by EGR1 in response to ionizing radiation include TNFα, P53, RB and BAX (Ahmed, 2004). |  |  |
|  |  | 227404_s_at |  |  |  |  |  |  |  |  |  |  |  |  |  |  |  |
| 10 | AXUD1 | 225557_at |  |  |  |  |  |  |  |  |  |  |  |  | AXUD1 interacts with AXIN1 to induce apoptosis (Ishigu*ro et* al, 2001). |  |  |
| 11 | ADM | 202912_at |  |  |  |  |  |  |  |  |  |  |  |  | ADM is anti-apoptotic through the PI3K/AKT pathway (Okumu*ra et* al, 2004). |  |  |
| 12 | SOX9 | 202936_s_at |  |  |  |  |  |  |  |  |  |  |  |  | SOX9 increases apoptosis by inhibiting cell growth and G0/G1 cycle arrest (Drivda*hl et* al, 2004). |  |  |
| 13 | CSPG2 | 204620_s_at |  |  |  |  |  |  |  |  |  |  |  |  | Cells expressing CSPG2 (versican) are resistant to free radical induced apoptosis (*Wu et* al, 2002). |  |  |
|  |  | 221731_x_at |  |  |  |  |  |  |  |  |  |  |  |  |  |  |  |
|  |  | 211571_s_at |  |  |  |  |  |  |  |  |  |  |  |  |  |  |  |
|  |  | 215646_s_at |  |  |  |  |  |  |  |  |  |  |  |  |  |  |  |
| 14 | NOTCH2 | 212377_s_at |  |  |  |  |  |  |  |  |  |  |  |  | NOTCH2 targets CD23A, over expressed in tumours (Hubma*nn et* al, 2002, Linenberg*er et* al, 1999). |  |  |
| 15 | CD47 | 211075_s_at |  |  |  |  |  |  |  |  |  |  |  |  | CD47 mediates killing of breast cancer cells via a novel pathway involving regulation of cAMP levels by heterotrimeric Gi with subsequent effects mediated by PKA (Manna and Frazer, 2004). |  |  |
|  |  | 213857_s_at |  |  |  |  |  |  |  |  |  |  |  |  |  |  |  |
| 16 | NKX2-5 | 206578_at |  |  |  |  |  |  |  |  |  |  |  |  | NKX2-5 mutant mice have enhanced apoptosis in embryos (Tana*ka et* al, 2001). |  |  |
| 17 | SIVA | 210792_x_at |  |  |  |  |  |  |  |  |  |  |  |  | SIVA is a pro-apoptotic protein, a direct target for tumour suppressors P53 and E2F1 (Fort*in et* al, 2004). |  |  |
|  |  | 203489_at |  |  |  |  |  |  |  |  |  |  |  |  |  |  |  |
| 18 | RNF7 | 224439_x_at |  |  |  |  |  |  |  |  |  |  |  |  | Protects apoptosis through inhibition of cytochrome c release/caspase activation (S*un et* al, 2001). |  |  |
|  |  | 224395_s_at |  |  |  |  |  |  |  |  |  |  |  |  |  |  |  |
|  |  | 218286_s_at |  |  |  |  |  |  |  |  |  |  |  |  |  |  |  |
| 19 | DAF | 201926_s_at |  |  |  |  |  |  |  |  |  |  |  |  | DAF protects normal tissues from accidental injury by activating complement. The drug rituximab, an antibody to DAF increases apoptosis in tumours (Fishels*on et* al, 2003, Gol*ay et* al, 2000). |  |  |
|  |  | 201925_s_at |  |  |  |  |  |  |  |  |  |  |  |  |  |  |  |
| 20 | CD9 | 201005_at |  |  |  |  |  |  |  |  |  |  |  |  | CD9 ab. inhibits cell proliferation, causes apoptosis via JUN/MAPK/CASP3. (Muraya*ma et* al, 2004). |  |  |
| 21 | CD14 | 209224_s_at |  |  |  |  |  |  |  |  |  |  |  |  | CD14 antibody induces differentiation and apoptosis of AML cells, inhibits MYC. (So*ng et* al, 2004). |  |  |
| 22 | TEGT | 200803_s_at |  |  |  |  |  |  |  |  |  |  |  |  | TEGT is an inhibitor of apoptosis – suppresses BAX expression. (Je*an et* al, 1999). |  |  |
|  |  | 200804_at |  |  |  |  |  |  |  |  |  |  |  |  |  |  |  |
| 23 | ZNF313 | 200868_s_at |  |  |  |  |  |  |  |  |  |  |  |  | No information. Original paper (*Ma et* al, 2003). |  |  |
| 24 | FKBP2 | 203391_at |  |  |  |  |  |  |  |  |  |  |  |  | FKBP2 accelerates protein folding (Padil*la et* al, 2003). |  |  |
| 25 | MADH7 | 204790_at |  |  |  |  |  |  |  |  |  |  |  |  | MADH7 is anti-apoptotic, enhances NFKB and survival (Arno*ld et* al, 2004). |  |  |
| 26 | GATA6 | 210002_at |  |  |  |  |  |  |  |  |  |  |  |  | GATA6 may be important regulating /proliferation (Suzu*ki et* al, 1996, Hugg*on et* al, 1997). |  |  |
| 27 | TNFRSF12A | 218368_s_at |  |  |  |  |  |  |  |  |  |  |  |  | TWEAK receptor, increases neurite growth (Tana*be et* al, 2003, Pol*ek et* al, 2003). |  |  |
| 28 | IRF3 | 202621_at |  |  |  |  |  |  |  |  |  |  |  |  | IRF3/cAMP caused DNA damage (K*im et* al, 1999). IRF3 mutant could be oncogenic (K*im et* al, 2003). |  |  |
| 29 | ANAPC2 | 218555_at |  |  |  |  |  |  |  |  |  |  |  |  | No apoptotic information. ANAPC2 is part of anaphase-promoting complex (Naga*se et* al, 2000). |  |  |
| 30 | JUNB | 203022_at |  |  |  |  |  |  |  |  |  |  |  |  | JUNB null mice show proliferation (Passeg*ue et* al, 2002). Under NFKB control (Math*as et* al, 2002). |  |  |
| 31 | DIPA | 204610_s_at |  |  |  |  |  |  |  |  |  |  |  |  | DIPA is pathogenic human virus, causes disease with hepatitis B virus (Brazas and Ganem, 1996). |  |  |
| 32 | PTP4A1 | 200732_s_at |  |  |  |  |  |  |  |  |  |  |  |  | Over-expression of PTP4A1 induces metastatic tumour formation and resistance to apoptosis (Ze*ng et* al, 2003). |  |  |
|  |  | 200731_s_at |  |  |  |  |  |  |  |  |  |  |  |  |  |  |  |
| 33 | CD59 | 200983_x_at |  |  |  |  |  |  |  |  |  |  |  |  | CD59 prevents Membrane Attack Complex (MAC) forming, MAC blasts holes in membrane and causes apoptosis, via PI3K/BAD/BCL2/MAPK/CASP3 (Fishelson, et al., 2003, Cole and Morgan, 2003). |  |  |
|  |  | 200985_s_at |  |  |  |  |  |  |  |  |  |  |  |  |  |  |  |
| 34 | BIRC5 | 210334_x_at |  |  |  |  |  |  |  |  |  |  |  |  | BIRC5 is an inhibitor of apoptosis, may counteract a default induction of apoptosis in G2/M phase. Inhibits CASP3 and 7. (*Li et* al, 1998) |  |  |
|  |  | 202094_at |  |  |  |  |  |  |  |  |  |  |  |  |  |  |  |
|  |  | 202095_s_at |  |  |  |  |  |  |  |  |  |  |  |  |  |  |  |
|  |  | 1555826_at |  |  |  |  |  |  |  |  |  |  |  |  |  |  |  |
| 35 | PORIMIN | 211967_at |  |  |  |  |  |  |  |  |  |  |  |  | Pro-oncosis receptor induces membrane injury, causes death similar to apoptosis (Zha*ng et* al, 1998). |  |  |
| 36 | SIRT2 | 220605_s_at |  |  |  |  |  |  |  |  |  |  |  |  | Similar sequence SIRT1. (Frye, 1999) Inactive SIRT1 induces apoptosis through p53 (L*uo et* al, 2001). |  |  |
| 37 | SON | 226465_s_at |  |  |  |  |  |  |  |  |  |  |  |  | SON has sequence similarities to oncogenes of the MYC family (Berdichevsk*ii et* al, 1988). |  |  |
| 38 | APLP2 | 228520_s_at |  |  |  |  |  |  |  |  |  |  |  |  | APP produces ABETA and AID which lower cell threshold to apoptosis and repress NOTCH, cleaved APLP2 acts in similar way to AID through CASP3 and 9 (Scheinfeld, et al., 2002, Cowan, et al., 2001). |  |  |
|  |  | 208702_x_at |  |  |  |  |  |  |  |  |  |  |  |  |  |  |  |
| 39 | SIRT1 | 218878_s_at |  |  |  |  |  |  |  |  |  |  |  |  | Inactive SIRT1 induces apoptosis through P53 (Luo, et al., 2001). |  |  |
| 40 | YARS | 238760_at |  |  |  |  |  |  |  |  |  |  |  |  | YARS fragments have cytokine acitivity, may signal macrophages (Weiner and Maizels, 1999). |  |  |
| 41 | CDC2L1 | 211289_x_at |  |  |  |  |  |  |  |  |  |  |  |  | CDC2L1 isoforms are 58-110kD. P58 found in cells undergoing apoptosis (Xia*ng et* al, 1994). |  |  |
| 42 | CDC2L2 | 207428_x_at |  |  |  |  |  |  |  |  |  |  |  |  | CDC2L2 isoforms are 65-110kD. P110 found in cells undergoing apoptosis (Xiang, et al., 1994). |  |  |
| 43 | BAG1 | 211475_s_at |  |  |  |  |  |  |  |  |  |  |  |  | BAG1 prevented apoptosis in serum deprived cells, associated with BCL2 (Takayama, et al., 1995). |  |  |
| 44 | TYK2 | 205546_s_at |  |  |  |  |  |  |  |  |  |  |  |  | TYK2 induces DAXX which causes apoptosis (Muromo*to et* al, 2004, Muromo*to et* al, 2003). |  |  |
| 45 | DFFA | 203277_at |  |  |  |  |  |  |  |  |  |  |  |  | DFFA induces DNA fragmentation after it is activated by CASP3 (L*iu et* al, 1997). |  |  |
|  |  | 223518_at |  |  |  |  |  |  |  |  |  |  |  |  |  |  |  |
| 46 | BSG | 208677_s_at |  |  |  |  |  |  |  |  |  |  |  |  | No information on apoptosis. Plays a role in intercellular recognition. (Kaneku*ra et* al, 1991) |  |  |
| 47 | JUN | 201465_s_at |  |  |  |  |  |  |  |  |  |  |  |  | Active JNK phosphorylates the transcription factor JUN which is crucial for the induction of apoptosis. (Wilson, 2002) |  |  |
|  |  | 201466_s_at |  |  |  |  |  |  |  |  |  |  |  |  |  |  |  |
| 48 | ARL3 | 202641_at |  |  |  |  |  |  |  |  |  |  |  |  | No apoptotic information. GTP-binding protein, RAS superfamily. (Cavena*gh et* al, 1994) |  |  |
| 49 | CDC42 | 208727_s_at |  |  |  |  |  |  |  |  |  |  |  |  | CDC42 is ivolved in interplay of small GTPase proteins of Ras superfamily (rac, rho, CDC42) re-model actin in tumors. (Rao and Li, 2004) |  |  |
|  |  | 208728_s_at |  |  |  |  |  |  |  |  |  |  |  |  |  |  |  |
| 50 | CDK5 | 204247_s_at |  |  |  |  |  |  |  |  |  |  |  |  | P52 decreases CDK5, neuron apoptosis Alzheimers (Nguy*en et* al, 2002, Monaco and Vallano, 2003) |  |  |
| 51 | HTATIP | 206689_x_at |  |  |  |  |  |  |  |  |  |  |  |  | HTATIP acetylates the androgen receptor which regulates growth properties of cells. Androgen receptor mutants reduce apoptosis (*Fu et* al, 2004). |  |  |
|  |  | 209192_x_at |  |  |  |  |  |  |  |  |  |  |  |  |  |  |  |
|  |  | 214258_x_at |  |  |  |  |  |  |  |  |  |  |  |  |  |  |  |
| 52 | PTPN13 | 204201_s_at |  |  |  |  |  |  |  |  |  |  |  |  | PTPN13 inhibits FAS-induced apoptosis (Iniza*wa et* al, 1996). |  |  |
| 53 | PTP4A2 | 208615_s_at |  |  |  |  |  |  |  |  |  |  |  |  | PTP4A2 over-expression induces tumour growth (Cat*es et* al, 1996). |  |  |
|  |  | 208617_s_at |  |  |  |  |  |  |  |  |  |  |  |  |  |  |  |
| 54 | FOXO3A | 210655_s_at |  |  |  |  |  |  |  |  |  |  |  |  | When unbound from AKT1 triggers apoptosis by inducing genes such as TNFSF6 (Brun*et et* al, 2004). |  |  |
| 55 | UTRN | 225093_at |  |  |  |  |  |  |  |  |  |  |  |  | CASP8 targeted BCAP31 recruits UTRN and mitochondria sciession (Brekenrid*ge et* al, 2003). |  |  |
| 56 | EMP3 | 203729_at |  |  |  |  |  |  |  |  |  |  |  |  | EMP3 regulates cell proliferation/apoptosis (Jetten and Suter, 2000, Taylor and Suter, 1996). |  |  |
| 57 | RAD21 | 200608_s_at |  |  |  |  |  |  |  |  |  |  |  |  | RAD21 cleaved by CASP3/7, possible amplifier of apoptotic signal (Pa*ti et* al, 2002, Ch*en et* al, 2002). |  |  |
| 58 | PDCD5 | 219275_at |  |  |  |  |  |  |  |  |  |  |  |  | PDCD5 is up-regulated in tumour cells undergoing apoptosis (L*iu et* al, 2003). |  |  |
| 59 | DDX41 | 217840_at |  |  |  |  |  |  |  |  |  |  |  |  | Essential for survival, mutant DDX41 flies had specific defects in apoptosis (Irion and Leptin, 1999). |  |  |
| 60 | PLAGL1 | 207943_x_at |  |  |  |  |  |  |  |  |  |  |  |  | PLAGL1 inhibits tumour cell proliferation in nude mice, same pathway as p53 (Spengl*er et* al, 1997). |  |  |
| 61 | ILF3 | 217804_s_at |  |  |  |  |  |  |  |  |  |  |  |  | No apoptotic information. May act as a translation inhibitory protein (Xu and Grabowski, 1999). |  |  |
|  |  | 208930_s_at I |  |  |  |  |  |  |  |  |  |  |  |  |  |  |  |
| 62 | IFNGR1 | 211676_s_at |  |  |  |  |  |  |  |  |  |  |  |  | IFNGR1 antibody causes decreased apoptosis (Takahas*hi et* al, 2001). |  |  |
| 63 | DIABLO | 219350_s_at |  |  |  |  |  |  |  |  |  |  |  |  | DIABLO promotes CASP9, removes inhibitory activity of IAPs (*Du et* al, 2000, Verhag*en et* al, 2000). |  |  |
| 64 | ATF3 | 202672_s_at |  |  |  |  |  |  |  |  |  |  |  |  | ATF3 stress induced apoptosis regulator, NFKB/JUN mediated (Harm*an et* al, 2004, Ba*ek et* al, 2004). |  |  |
| 65 | MAP2K2 | 213490_s_at |  |  |  |  |  |  |  |  |  |  |  |  | Increases apoptosis through activation of RAF1/MEK/ERK (Plesch*ka et* al, 2001) and JNK/p38/NFkB pathways (Or*th et* al, 1999). |  |  |
|  |  | 202424_at |  |  |  |  |  |  |  |  |  |  |  |  |  |  |  |
| 66 | SSA2 | 210438_x_at |  |  |  |  |  |  |  |  |  |  |  |  | SSA2 is found in apoptotic blebs (Ohlss*en et* al, 2002). Has also been shown in UVA-irradiated keratinocytes the distribution of SSA2 is the same as FASL and BAX (Bollain-y-Goyt*ia et* al, 2000). |  |  |
|  |  | 212852_s_at |  |  |  |  |  |  |  |  |  |  |  |  |  |  |  |
| 67 | PPP1CA | 200846_s_at |  |  |  |  |  |  |  |  |  |  |  |  | No apoptotic information. PPP1CA is essential for cell division (Veech, 2003). |  |  |
| 68 | PTPRF | 200637_s_at |  |  |  |  |  |  |  |  |  |  |  |  | PTPRF signals CASP3 cleavage (Ho*on et* al, 2003), down-regulated tumour tissue (Liu, et al., 2003). |  |  |
| 69 | MAPK8IP3 | 213178_s_at |  |  |  |  |  |  |  |  |  |  |  |  | MAPK8IP3 binds ASK1 and enhances JNK activity via SEK1/MKK4 (Matsuu*ra et* al, 2002). |  |  |
| 70 | MIF | 1556316_s_at |  |  |  |  |  |  |  |  |  |  |  |  | Delays BID/BAX cleavage, stops cytochrome/DIABLOrelease/CASP3 activation (Bauma*nn et* al, 2003) |  |  |
| 71 | APP | 200602_at |  |  |  |  |  |  |  |  |  |  |  |  | APP is directly and efficiently cleaved by caspases (mostly CASP3) during apoptosis resulting in elevated amyloid beta formation (Gerva*is et* al, 1999). |  |  |
|  |  | 214953_s_at |  |  |  |  |  |  |  |  |  |  |  |  |  |  |  |
| 72 | BCAP31 | 200837_at |  |  |  |  |  |  |  |  |  |  |  |  | Activates BCAP31, mitochondral ca. mechanism (Chandra, et al., 2004, Breckenridge, et al., 2003). |  |  |
| 73 | HIF1A | 200989_at |  |  |  |  |  |  |  |  |  |  |  |  | HIF1A activates NIP3, which primes cells for apoptosis when oxygen deprived (Bruick, 2000). |  |  |
| 74 | PDAP1 | 202290_at |  |  |  |  |  |  |  |  |  |  |  |  | PDAP1 enhanced PDGFA-stimulated cell growth in mouse fibroblasts (Fischer and Schubert, 1996). |  |  |
| 75 | DAPK1 | 203139_at |  |  |  |  |  |  |  |  |  |  |  |  | DAPK1 is a positive mediator of gamma-interferon induced apoptosis (Dei*ss et* al, 1995). |  |  |
| 76 | PDCD6 | 203415_at |  |  |  |  |  |  |  |  |  |  |  |  | PDCD6 is required for Fas, T-cell receptor and glucocorticoid induced death (Vi*to et* al, 1996). |  |  |
| 77 | MID1 | 203636_at |  |  |  |  |  |  |  |  |  |  |  |  | No information on apoptosis. A defect in MID1 causes midline abnormalities such as cleft lip and is known as Opitz syndrome (Opitz, 1987). |  |  |
|  |  | 203637_s_at |  |  |  |  |  |  |  |  |  |  |  |  |  |  |  |
| 78 | GADD45A | 203725_at |  |  |  |  |  |  |  |  |  |  |  |  | GADD45A activates MTK1 which activates p38, JNK and apoptosis (Takekawa and Saito, 1998). |  |  |
| 79 | CASP7 | 207181_s_at |  |  |  |  |  |  |  |  |  |  |  |  | CASP7 is a downstream executioner caspase. (Barrett and Rawlings, 2001). |  |  |
| 80 | DP1 | 208872_s_at |  |  |  |  |  |  |  |  |  |  |  |  | DP1 binds transcription factor E2F which promotes apoptosis through P53 and also through P73, APAF1 and BAX. DP1 also inhibits NFKB signal (Hitchens and Robbins, 2003). |  |  |
|  |  | 208873_s_at |  |  |  |  |  |  |  |  |  |  |  |  |  |  |  |
| 81 | FOS | 209189_at |  |  |  |  |  |  |  |  |  |  |  |  | FOS, JUN and subsequently AP1 over-expression, induces apoptosis (Suomalain*en et* al, 2004). |  |  |
| 82 | PBP | 211941_s_at |  |  |  |  |  |  |  |  |  |  |  |  | PBP is a RAF kinase inhibitor protein. Loss of RKIP protects against apoptosis (Keller, 2004). |  |  |
| 83 | MAP2K3 | 215498_s_at |  |  |  |  |  |  |  |  |  |  |  |  | MAP2K3 binds YOPJ increases apoptosis - inhibits ERK/JNK/P38/NFKB pathways (Orth, et al., 1999). |  |  |
| 84 | PERP | 217744_s_at |  |  |  |  |  |  |  |  |  |  |  |  | PERP is highly expressed in genes undergoing P53 apoptosis. PERP targets p53 (Ihr*ie et* al, 2003). |  |  |
| 85 | GPS1 | 217782_s_at |  |  |  |  |  |  |  |  |  |  |  |  | GPS1 over-expression suppresses RAS and JNK activity (Spa*in et* al, 1996). |  |  |
| 86 | SAV1 | 218276_s_at |  |  |  |  |  |  |  |  |  |  |  |  | SAV1 promotes cell cycle exit and cell death (Tap*on et* al, 2002). |  |  |
| 87 | P14 | 223416_at |  |  |  |  |  |  |  |  |  |  |  |  | Mutant P14 has been observed in tumours (K*im et* al, 2004). |  |  |
| 88 | MADH5 | 225223_at |  |  |  |  |  |  |  |  |  |  |  |  | SMAD5 RNA interference completely inhibited H.pylor-induced apoptosis (Nagas*ko et* al, 2003). |  |  |
| 89 | HIPK2 | 225368_at |  |  |  |  |  |  |  |  |  |  |  |  | HIPK2 enhances expression of P53 target genes (Hoffma*nn et* al, 2002). |  |  |
| 90 | FZD8 | 227405_s_at |  |  |  |  |  |  |  |  |  |  |  |  | Human FZD8 may activate CTNNB1-TCF signalling pathway (Sait*oh et* al, 2001). |  |  |
| 91 | BIRC4 | 228363_at |  |  |  |  |  |  |  |  |  |  |  |  | BIRC4 directly inhibits CASP3, 7 (Devere*ux et* al, 1997) and 9 (Srinivasu*la et* al, 2001). |  |  |
| 92 | PVRL2 | 232078_at |  |  |  |  |  |  |  |  |  |  |  |  | No apoptotic information. Receptor for HSV1 and HSV2 entry into cells (Eber*le et* al, 1995). |  |  |
|  |  | 203149_at |  |  |  |  |  |  |  |  |  |  |  |  |  |  |  |
|  |  | 232079_s_at |  |  |  |  |  |  |  |  |  |  |  |  |  |  |  |
| 93 | DAPK3 | 203890_s_at |  |  |  |  |  |  |  |  |  |  |  |  | DAPK3 over-expression induced apoptotic changes in mammalian cells (Kaw*ai et* al, 1998). |  |  |
| 94 | NME6 | 205851_at |  |  |  |  |  |  |  |  |  |  |  |  | NME6 is an inhibitor of P53 induced apoptosis (Mehus and Deloukas, 1999) (Nakamu*ra et* al, 1997). |  |  |
| 95 | PTPRS | 226571_s_at |  |  |  |  |  |  |  |  |  |  |  |  | No apoptotic information. PTPRS is a member of a subfamily of receptor type PTPases. (Puli*do et* al, 1995) |  |  |
|  |  | 229465_s_at |  |  |  |  |  |  |  |  |  |  |  |  |  |  |  |
| 96 | LRDD | 221640_s_at |  |  |  |  |  |  |  |  |  |  |  |  | LRDD is induced by P53 and promotes apoptosis (L*in et* al, 2000). |  |  |
| 97 | ZRANB1 | 201219_at |  |  |  |  |  |  |  |  |  |  |  |  | No apoptotic information. Modulates TNFa signaling (Eva*ns et* al, 617-623). |  |  |
| 98 | RALBP1 | 202844_s_at |  |  |  |  |  |  |  |  |  |  |  |  | No apoptotic information. RALBP1 interacts with GTPases RALA and B (Jullien-Flor*es et* al, 1995). |  |  |
| 99 | TRAF4 | 202871_at |  |  |  |  |  |  |  |  |  |  |  |  | TRAF4 is over expressed in breast carcinoma. Appears to be involved in TNF-related cytokine signal transduction (Regni*er et* al, 2002). |  |  |
|  |  | 206837_at |  |  |  |  |  |  |  |  |  |  |  |  |  |  |  |
| 100 | BAD | 209364_at |  |  |  |  |  |  |  |  |  |  |  |  | BAD binds BClX, displaces BAX and restores apoptotic ability (Yang, et al., 1995). |  |  |
| 101 | FYN | 216033_s_at |  |  |  |  |  |  |  |  |  |  |  |  | FYN null mice impair NFKB and B-cell activity, may impair survival response (Sai*jo et* al, 2003). |  |  |
| 102 | MSX2 | 205555_s_at |  |  |  |  |  |  |  |  |  |  |  |  | MSX2 mutant mice malformations from teratogen exposure cause cell death. (Winogr*ad et* al, 1997). |  |  |
| 103 | JAK1 | 1552611_a_at |  |  |  |  |  |  |  |  |  |  |  |  | JAK1 activates STAT3 which is found over expressed in many cancers (Bart*on et* al, 2004). |  |  |
| 104 | NAP1L4 | 1560339_s_at |  |  |  |  |  |  |  |  |  |  |  |  | No apoptotic information. Belongs to the nucleosome assembly protein (NAP) family (*Hu et* al, 1996). |  |  |
| 105 | RAC1 | 1567458_s_at |  |  |  |  |  |  |  |  |  |  |  |  | Small GTPase, regulates phagocytosis of apoptotic cells (Leverrier and Ridley, 2001). |  |  |
| 106 | DAD1 | 200046_at |  |  |  |  |  |  |  |  |  |  |  |  | In a temperature sensitive cell line, loss of DAD-1 protein triggered apoptosis (Nakashi*ma et* al, 1993). |  |  |
| 107 | STAT1 | 200887_s_at |  |  |  |  |  |  |  |  |  |  |  |  | STAT1 deficient mice have neurodegeneration/inflammation/ increased apoptosis (Wa*ng et* al, 2002). |  |  |
| 108 | SGK | 201739_at |  |  |  |  |  |  |  |  |  |  |  |  | SGK activates the glucocorticoid receptor which has an anti-apoptotic effect (Miko*sz et* al, 2001). |  |  |
| 109 | DAXX | 201763_s_at |  |  |  |  |  |  |  |  |  |  |  |  | DAXX binds to the FAS death domain and enhances FAS-mediated apoptosis (Ya*ng et* al, 1997). |  |  |
| 110 | RNF14 | 201824_at |  |  |  |  |  |  |  |  |  |  |  |  | No apoptotic information. RNFs mediate protein-DNA/protein interactions (Ue*ki et* al, 1999). |  |  |
| 111 | ARIH1 | 201879_at |  |  |  |  |  |  |  |  |  |  |  |  | No apoptotic information. Involved in ubiquitination (Moynih*an et* al, 1999). |  |  |
| 112 | ITGAV | 202351_at |  |  |  |  |  |  |  |  |  |  |  |  | No apoptotic information. ITGAV is an alpha-V integrin (Suzu*ki et* al, 1986). |  |  |
| 113 | TIEG | 202393_s_at |  |  |  |  |  |  |  |  |  |  |  |  | TIEG over-expression in TGFB sensitive epithelial cell line induced apoptosis (Tachiba*na et* al, 1997). |  |  |
| 114 | MAP4K5 | 203552_at |  |  |  |  |  |  |  |  |  |  |  |  | No apoptotic information. MAP4K5 has kinase activity and activates JNK (Tung and Blenis, 1997). |  |  |
| 115 | E2F3 | 203693_s_at |  |  |  |  |  |  |  |  |  |  |  |  | E2F3 suppresses tumour suppressor ARF, imp. In P53 tumour surveillance pathway (Ginsberg, 2004). |  |  |
| 116 | JUND | 203752_s_at |  |  |  |  |  |  |  |  |  |  |  |  | JUND is a P53 modulator, protects from cell stress induced apoptosis (Weitzm*an et* al, 2000). |  |  |
| 117 | GULP1 | 204237_at |  |  |  |  |  |  |  |  |  |  |  |  | GULP1 engulfs phagocytic cells, it has been linked to CD91/LRP/CED1 (*Su et* al, 2002). |  |  |
| 118 | CD151 | 204306_s_at |  |  |  |  |  |  |  |  |  |  |  |  | No apoptotic information. CD151 is a transmembrane 4 superfamily protein member (Fitt*er et* al, 1995). |  |  |
| 119 | PDGFA | 205463_s_at |  |  |  |  |  |  |  |  |  |  |  |  | No apoptotic information. Platelet derived growth factor A (Betshol*tz et* al, 1986). |  |  |
| 120 | AMH | 206516_at |  |  |  |  |  |  |  |  |  |  |  |  | AMH-mediated NFKB pathway used in cancer cells G1 arrest and apoptosis (Donah*oe et* al, 2003). |  |  |
| 121 | CREM | 207630_s_at |  |  |  |  |  |  |  |  |  |  |  |  | CREM is rapidly upregulated by beta-adrenergic receptor in cardiac myocytes and works as a negative regulator of hypertrophy as well as a positive mediator of apoptosi. (Tomi*ta et* al, 2001). |  |  |
|  |  | 214508_x_at |  |  |  |  |  |  |  |  |  |  |  |  |  |  |  |
| 122 | RBPSUH | 207785_s_at |  |  |  |  |  |  |  |  |  |  |  |  | Interacts with NOTCH 1,2 and 3. Involved broad spectrum cell fate decisions (Callah*an et* al, 2000). |  |  |
| 123 | PSMD11 | 208777_s_at |  |  |  |  |  |  |  |  |  |  |  |  | No apoptotic information. PSMD11 involved degradation of ubiquitinated proteins (Sai*to et* al, 1997). |  |  |
| 124 | TNFRSF10B | 209295_at |  |  |  |  |  |  |  |  |  |  |  |  | Over-expression induces caspase dependent apoptotic pathway via FADD (Walcz*ak et* al, 1997). |  |  |
| 125 | MAX | 209332_s_at |  |  |  |  |  |  |  |  |  |  |  |  | FASL potent inducer of apoptosis. activity increased when MAX over expressed (Wien*er et* al, 2004). |  |  |
| 126 | CHUK | 209666_s_at |  |  |  |  |  |  |  |  |  |  |  |  | CHUK null mice high apoptosis B cells, little P52/lots P100, defective NFKB2 (Sentfleb*en et* al, 2001). |  |  |
| 127 | PBEF1 | 217739_s_at |  |  |  |  |  |  |  |  |  |  |  |  | PBEF1 removal causes apoptosis by IL-1B, GCSF, IL8 and TNFA. CASP8/3 involved (J*ia et* al, 2004). |  |  |
| 128 | PDGFC | 218718_at |  |  |  |  |  |  |  |  |  |  |  |  | No apoptotic information. Platelet derived growth factor, motility stiumulant (Reigst*ad et* al, 2003). |  |  |
| 129 | CBL | 225231_at |  |  |  |  |  |  |  |  |  |  |  |  | CBL reduces ubiquitylation/degradation BIM, pro-apoptotic BCL2 family member (Akiya*ma et* al, 2003). |  |  |
| 130 | MAPK8 | 226046_at |  |  |  |  |  |  |  |  |  |  |  |  | MAPK8 is also called JNK. It is required for UV-induced apoptosis in primary murine embryonic fibroblasts. (Tourni*er et* al, 2000) |  |  |
|  |  | 226048_at |  |  |  |  |  |  |  |  |  |  |  |  |  |  |  |

Table 3. Families and gene interactors in Apoptosis Differentials List (some genes are included from the Differentials List).

Protein tyrosine phosphatase (PTP) family members

| **NO.** | **GENE NAME** | **PPROBE** | **A**  **12** | **A**  **24** | **A**  **48C4** | **SK12** | **SK24** | **SK48** | **XB12** | **XB24** | **XB48** | **SS12** | **SS24** | **SS48** | **FUNCTION AND REFERENCE** | **APOP** | **EXPT** |
| --- | --- | --- | --- | --- | --- | --- | --- | --- | --- | --- | --- | --- | --- | --- | --- | --- | --- |
| 52 | PTPN13 | 204201_s_at |  |  |  |  |  |  |  |  |  |  |  |  | PTPN13 inhibits FAS-induced apoptosis (Inizawa, et al., 1996). |  |  |
| 53 | PTP4A2 | 208615_s_at |  |  |  |  |  |  |  |  |  |  |  |  | PTP4A2 over-expression induces tumour growth (Cates, et al., 1996). |  |  |
|  |  | 208617_s_at |  |  |  |  |  |  |  |  |  |  |  |  |  |  |  |
| 32 | PTP4A1 | 200732_s_at |  |  |  |  |  |  |  |  |  |  |  |  | Over-expression of PTP4A1 induces metastatic tumour formation and resistance to apoptosis (Zeng, et al., 2003). |  |  |
|  |  | 200731_s_at |  |  |  |  |  |  |  |  |  |  |  |  |  |  |  |
| 67 | PPP1CA | 200846_s_at |  |  |  |  |  |  |  |  |  |  |  |  | No apoptotic information. PPP1CA is essential for cell division (Veech, 2003). |  |  |
| 68 | PTPRF | 200637_s_at |  |  |  |  |  |  |  |  |  |  |  |  | PTPRF signals CASP3 cleavage (Hoon, et al., 2003), down-regulated tumour tissue (Liu, et al., 2003). |  |  |
| 95 | PTPRS | 226571_s_at |  |  |  |  |  |  |  |  |  |  |  |  | No apoptotic information. PTPRS is a member of a subfamily of receptor type PTPases. (Pulido, et al., 1995) |  |  |
|  |  | 229465_s_at |  |  |  |  |  |  |  |  |  |  |  |  |  |  |  |

Frizzled (FZD) family members

| 4 | FZD7 | 203706_s_at |  |  |  |  |  |  |  |  |  |  |  |  | FZD encode WNT proteins which act through TCF, JNK and calcium pathways (Kirikoshi and Katoh, 2002, Herin and Sheng, 2002). |  |  |
| --- | --- | --- | --- | --- | --- | --- | --- | --- | --- | --- | --- | --- | --- | --- | --- | --- | --- |
|  |  | 203705_s_at |  |  |  |  |  |  |  |  |  |  |  |  |  |  |  |
| 90 | FZD8 | 227405_s_at |  |  |  |  |  |  |  |  |  |  |  |  | Human FZD8 may activate CTNNB1-TCF signalling pathway (Saitoh, et al., 2001). |  |  |
|  |  |  |  |  |  |  |  |  |  |  |  |  |  |  | Differentials Lists |  |  |

BCL2 family members

| 6 | BAX | 211833_s_at |  |  |  |  |  |  |  |  |  |  |  |  | BAX binds and antagonises BCL2 and causes apoptosis via CASP3 (Willis, et al., 2003). |  |  |
| --- | --- | --- | --- | --- | --- | --- | --- | --- | --- | --- | --- | --- | --- | --- | --- | --- | --- |
|  |  | 208478_s_at |  |  |  |  |  |  |  |  |  |  |  |  |  |  |  |
| 43 | BAG1 | 211475_s_at |  |  |  |  |  |  |  |  |  |  |  |  | BAG1 prevented apoptosis in serum deprived cells, associated with BCL2 (Takayama, et al., 1995). |  |  |
| 100 | BAD | 209364_at |  |  |  |  |  |  |  |  |  |  |  |  | BAD binds BCLX, displaces BAX and restores apoptotic ability (Yang, et al., 1995). |  |  |

RING Finger (RNF) family members

| 110 | RNF14 | 201824_at |  |  |  |  |  |  |  |  |  |  |  |  | No apoptotic information. RNFs mediate protein-DNA/protein interactions (Ueki, et al., 1999). |  |  |
| --- | --- | --- | --- | --- | --- | --- | --- | --- | --- | --- | --- | --- | --- | --- | --- | --- | --- |
| 18 | RNF7 | 224439_x_at |  |  |  |  |  |  |  |  |  |  |  |  | Protects apoptosis through inhibition of cytochrome c release/caspase activation (Sun, et al., 2001). |  |  |
|  |  | 224395_s_at |  |  |  |  |  |  |  |  |  |  |  |  |  |  |  |
|  |  | 218286_s_at |  |  |  |  |  |  |  |  |  |  |  |  |  |  |  |
|  | RNF40  RNF40 | 239801_at |  |  |  |  |  |  |  |  |  |  |  |  | Differentials Lists |  |  |
|  |  | 206845_s_at |  |  |  |  |  |  |  |  |  |  |  |  |  |  |  |
|  | RNF8 | 203160_s_at |  |  |  |  |  |  |  |  |  |  |  |  | Differentials Lists |  |  |
|  | RNF126 | 223332_x_at |  |  |  |  |  |  |  |  |  |  |  |  | Differentials Lists |  |  |
|  |  | 215031_x_at |  |  |  |  |  |  |  |  |  |  |  |  |  |  |  |
|  |  | 205748_s_at |  |  |  |  |  |  |  |  |  |  |  |  |  |  |  |

Cell division control (CDC )family members

| 41 | CDC2L1 | 211289_x_at |  |  |  |  |  |  |  |  |  |  |  |  | CDC2L1 isoforms are 58-110kD. P58 found in cells undergoing apoptosis (Xiang, et al., 1994). |  |  |
| --- | --- | --- | --- | --- | --- | --- | --- | --- | --- | --- | --- | --- | --- | --- | --- | --- | --- |
| 42 | CDC2L2 | 207428_x_at |  |  |  |  |  |  |  |  |  |  |  |  | CDC2L2 isoforms are 65-110kD. P110 found in cells undergoing apoptosis (Xiang, et al., 1994). |  |  |
| 49 | CDC42 | 208727_s_at |  |  |  |  |  |  |  |  |  |  |  |  | CDC42 is ivolved in interplay of small GTPase proteins of Ras superfamily (rac, rho, CDC42) re-model actin in tumors. (Rao and Li, 2004) |  |  |
|  |  | 208728_s_at |  |  |  |  |  |  |  |  |  |  |  |  |  |  |  |
|  | CDC6 | 203967_at |  |  |  |  |  |  |  |  |  |  |  |  |  |  |  |
|  | CDC20 | 202870_s_at |  |  |  |  |  |  |  |  |  |  |  |  |  |  |  |
|  | CDC34 | 212540_at |  |  |  |  |  |  |  |  |  |  |  |  |  |  |  |
|  | CDC37 | 209953_s_at |  |  |  |  |  |  |  |  |  |  |  |  |  |  |  |
|  | CDC42EP1 | 204693_at |  |  |  |  |  |  |  |  |  |  |  |  |  |  |  |
|  | CDCA3 | 221436_s_at |  |  |  |  |  |  |  |  |  |  |  |  |  |  |  |
|  | CDC26 | 225422_at |  |  |  |  |  |  |  |  |  |  |  |  |  |  |  |
|  | CDC5L | 209057_x_at |  |  |  |  |  |  |  |  |  |  |  |  |  |  |  |
|  | CDC25A | 1555772_a_at |  |  |  |  |  |  |  |  |  |  |  |  |  |  |  |

MAPK family members

| **NO.** | **GENE NAME** | **A12** | **A24** | **A**  **48** | **SK12** | **SK24** | **SK48** | **XB12** | **XB24** | **XB48** | **SS12** | **SS24** | **SS48** | **FUNCTION AND REFERENCE** | **APOP** | **EXPT** |
| --- | --- | --- | --- | --- | --- | --- | --- | --- | --- | --- | --- | --- | --- | --- | --- | --- |
| 65 | 213490_s_at MAP2K2 |  |  |  |  |  |  |  |  |  |  |  |  | Increases apoptosis through activation of RAF1/MEK/ERK (Pleschka, et al., 2001) and JNK/p38/NFkB pathways (Orth, et al., 1999). |  |  |
|  | 202424_at MAP2K2 |  |  |  |  |  |  |  |  |  |  |  |  |  |  |  |
| 69 | 213178_s_at MAPK8IP3 |  |  |  |  |  |  |  |  |  |  |  |  | Binds ASK1 and enhances JNK activity via SEK1/MKK4 (Matsuura, et al., 2002). |  |  |
| 83 | 215498_s_at MAP2K3 |  |  |  |  |  |  |  |  |  |  |  |  | Binds YOPJ, increases apoptosis, inhibits ERK/JNK/p38/NFKB (Orth, et al., 1999). |  |  |
| 114 | 203552_at MAP4K5 |  |  |  |  |  |  |  |  |  |  |  |  | No apoptotic info. Has kinase activity and activates JNK (Tung and Blenis, 1997). |  |  |
| 130 | 226046_at MAPK8 |  |  |  |  |  |  |  |  |  |  |  |  | MAPK8 also called JNK. It is required for UV-induced apoptosis in primary murine embryonic fibroblasts (Tournier, et al., 2000). |  |  |
|  | 226048_at MAPK8 |  |  |  |  |  |  |  |  |  |  |  |  |  |  |  |

UTRN and BCAP31 interaction

| 55 | 225093_at UTRN |  |  |  |  |  |  |  |  |  |  |  |  | BCAP31 recruits UTRN, targets mitochondrial scission (Brekenridge, et al., 2003). |  |  |
| --- | --- | --- | --- | --- | --- | --- | --- | --- | --- | --- | --- | --- | --- | --- | --- | --- |
| 72 | 200837_at BCAP31 |  |  |  |  |  |  |  |  |  |  |  |  | CASP8 activates BCAP31, mitochondrial ca. mechanism (Chandra, et al., 2004, Breckenridge, et al., 2003). |  |  |

SIRT1 and FOXO family interaction

| 54 | 210655_s_at FOXO3A |  |  |  |  |  |  |  |  |  |  |  |  | Unbound from AKT1, triggers apoptosis, induces TNFSF6 (Brunet, et al., 2004). |  |  |
| --- | --- | --- | --- | --- | --- | --- | --- | --- | --- | --- | --- | --- | --- | --- | --- | --- |
| 36 | 220605_s_at SIRT2 |  |  |  |  |  |  |  |  |  |  |  |  | Similar sequence SIRT1 (Frye, 1999). |  |  |
| 39 | 218878_s_at SIRT1 |  |  |  |  |  |  |  |  |  |  |  |  | Inactive SIRT1 induces apoptosis through P53 (Luo, et al., 2001). |  |  |
| All | 203020_at HHL |  |  |  |  |  |  |  |  |  |  |  |  | Differentials List |  |  |

TNFR family members

| 27 | 218368_s_at TNFRSF12A |  |  |  |  |  |  |  |  |  |  |  |  | TWEAK receptor, works independently, increases neurites (Tanabe, et al., 2003, Polek, et al., 2003). |  |  |
| --- | --- | --- | --- | --- | --- | --- | --- | --- | --- | --- | --- | --- | --- | --- | --- | --- |
| 124 | 209295_at TNFRSF10B |  |  |  |  |  |  |  |  |  |  |  |  | Over- expression causes apoptosis via FADD (Walczak, et al., 1997). |  |  |

BIRC family members

| 34 | 210334_x_at BIRC5 |  |  |  |  |  |  |  |  |  |  |  |  | BIRC5 is an inhibitor of apoptosis, may counteract a default induction of apoptosis in G2/M phase. Inhibits CASP3 and 7 (Li, et al., 1998) . |  |  |
| --- | --- | --- | --- | --- | --- | --- | --- | --- | --- | --- | --- | --- | --- | --- | --- | --- |
|  | 202094_at BIRC5 |  |  |  |  |  |  |  |  |  |  |  |  |  |  |  |
|  | 202095_s_at BIRC5 |  |  |  |  |  |  |  |  |  |  |  |  |  |  |  |
|  | 1555826_at BIRC5 |  |  |  |  |  |  |  |  |  |  |  |  |  |  |  |
| 91 | 228363_at BIRC4 |  |  |  |  |  |  |  |  |  |  |  |  | Directly inhibits CASP3, 7 (Devereux, et al., 1997) and 9 (Srinivasula, et al., 2001). |  |  |

Alzheimer/NOTCH signaling

| 38 | 228520_s_at APLP2 |  |  |  |  |  |  |  |  |  |  |  |  | APP produces ABETA and AID, lower cell threshold to apoptosis, repress NOTCH, cleaved APLP2 acts in same wau through CASP3/9 (Scheinfeld, et al., 2002, Cowan, et al., 2001). |  |  |
| --- | --- | --- | --- | --- | --- | --- | --- | --- | --- | --- | --- | --- | --- | --- | --- | --- |
|  | 208702_x_at APLP2 |  |  |  |  |  |  |  |  |  |  |  |  |  |  |  |
| 71 | 200602_at APP |  |  |  |  |  |  |  |  |  |  |  |  | APP is directly and efficiently cleaved by caspases (mostly CASP3) during apoptosis resulting in elevated amyloid beta formation. (Gervais, et al., 1999) |  |  |
|  | 214953_s_at APP |  |  |  |  |  |  |  |  |  |  |  |  |  |  |  |
| 50 | 204247_s_at CDK5 |  |  |  |  |  |  |  |  |  |  |  |  | Decreased truncated P52, neuron apoptosis Alzheimers (Nguyen, et al., 2002, Monaco and Vallano, 2003). |  |  |
| 14 | 212377_s_at NOTCH2 |  |  |  |  |  |  |  |  |  |  |  |  | NOTCH2 targets CD23a, over expressed in many tumors. (Hubmann, et al., 2002, Linenberger, et al., 1999). |  |  |
| 122 | 207785_s_at RBPSUH |  |  |  |  |  |  |  |  |  |  |  |  | Interacts with NOTCH 1/2/3, cell fate decisions (Callahan, et al., 2000). |  |  |

FOS and JUN family members

| 30 | 203022_at JUNB |  |  |  |  |  |  |  |  |  |  |  |  | Null mice increase cells (Passegue, et al., 2002). NFKB control (Mathas, et al., 2002). |  |  |
| --- | --- | --- | --- | --- | --- | --- | --- | --- | --- | --- | --- | --- | --- | --- | --- | --- |
| 47 | 201465_s_at JUN |  |  |  |  |  |  |  |  |  |  |  |  | Active JNK phosphorylates the transcription factor JUN which is crucial for the induction of apoptosis (Wilson, 2002). |  |  |
|  | 201466_s_at JUN |  |  |  |  |  |  |  |  |  |  |  |  |  |  |  |
| 116 | 203752_s_at JUND |  |  |  |  |  |  |  |  |  |  |  |  | Modulator of P53/RAS pathway, protects from apoptosis (Weitzman, et al., 2000). |  |  |
| 81 | 209189_at FOS |  |  |  |  |  |  |  |  |  |  |  |  | FOS, JUN and AP1 over-expression, induces apoptosis (Suomalainen, et al., 2004). |  |  |

Small GTPases

| 48 | 202641_at ARL3 |  |  |  |  |  |  |  |  |  |  |  |  | No apoptotic information. GTP-binding protein RAS superfamily (Cavenagh, et al., 1994). |  |  |
| --- | --- | --- | --- | --- | --- | --- | --- | --- | --- | --- | --- | --- | --- | --- | --- | --- |
| 49 | 208727_s_at CDC42 |  |  |  |  |  |  |  |  |  |  |  |  | CDC42 is ivolved in interplay of small GTPase proteins of RAS superfamily (RAC, RHO, CDC42) re-model actin in tumors. (Rao and Li, 2004) |  |  |
|  | 208728_s_at CDC42 |  |  |  |  |  |  |  |  |  |  |  |  |  |  |  |
| 105 | 1567458_s_at RAC1 |  |  |  |  |  |  |  |  |  |  |  |  | Small GTPase, regulates phagocytosis of apoptotic cells (Leverrier and Ridley, 2001). |  |  |

The following discussion aims to provide an explanation as to why these genes might be present in Apoptosis Differentials List by highlighting their known associations with cell survival and apoptosis.

The MAP kinase family had five members present within the Apoptosis Differentials List, MAP2K2, MAP2K3, MAPK8IP3, MAP4K5 and MAPK8 (JNK). The first four all target MAPK8 (JNK), but through slightly different pathways. MAP2K2 directly effects ERK and MAP2K3 binds YOPJ which subsequently acts on ERK (Orth et al. 1999; Zheng and Guan 1993). MAPK8IP3 acts directly on ASK1 via the SEK1/MKK4 pathway (Matsuura et al. 2002). MAP4K5 acts throught the GCKR/SAPK pathway (Tung and Blenis 1997).

There were two genes present from the TNF receptor superfamily – TNFRSF12A and TNFRSF10B. TNFRSF12A has been shown to increase growth (Polek et al. 2003; Tanabe et al. 2003), so if withdrawn (as it would appear to be in this experiment) it might enhance apoptosis. TNFRSF12A was observed to be down regulated in expression at the 12 hour time point only in each sample so could be an early activator of apoptosis, as also concluded from the GO Cell Death pathway analysis. TNFRSF10B over-expression instigates a caspase dependent apoptotic pathway via FADD (Walczak et al. 1997) and appears over-expressed in this experiment at 48 hours after STS treatment so could be a late indicator of apoptosis. Sabatini in the original reverse transfection cell-based microarray experiment observed that the cells on their reverse transfection array growing over TNFRSF10B appeared stressed (Ziauddin and Sabatini 2001).

Protein tyrosine phosphatase family members PTP4A1, PTP4A2, PTPN13, PTPRF and PTPRS were present in the Apoptosis Differentials List. Over-expression of PTP4A1 and PTP4A2 can cause tumour growth (Cates et al. 1996; Zeng et al. 2003), PTP4A1 expression is increased in this study, therefore potentially promoting cell survival, but PTP4A2 is decreased, therefore potentially increasing the balance towards apoptosis. PTPRF is down-regulated in tumour tissue (Liu et al. 2003) and is down-regulated in this study and is therefore potentially acting to decrease apoptosis. PTPN13 inhibits FAS-induced apoptosis (Inizawa et al. 1996) and is up-regulated in this study, therefore again potentially acting to decrease apoptosis.

Frizzled family members, FZD7 and FZD8 were both present in the apoptosis Differentials List and increased in expression at similar time points. Frizzled (FZD) genes encode WNT receptors which transduce WNT signals to the beta-catenin-TCF pathway, the JNK pathway or the Ca^2+^ pathway (Herin and Sheng 2002; Kirikoshi and Katoh 2002) TCF7L2 (from the Differentials Lists) targets FZD (Thorstensen and Lothe 2003) genes and is present at the same time points.

BIRC family members BIRC4 and BIRC5 were also observed to alter in their expression. BIRC5 is an inhibitor of apoptosis and may counteract a default induction of apoptosis in G2/M phase, it inhibits CASP3 and 7 (Li et al. 1998). It is decreased in this study, therefore potentially increasing apoptosis. BIRC4 is increased in this study, it directly inhibits CASP3, 7 (Devereux et al. 1997) and 9 (Srinivasula et al. 2001). BIRC5 may be down regulated and therefore increasing apoptosis to counteract the apoptotic inhibitory effect of BIRC4.

There were many RING finger proteins whose expression was observed to change in these studys. The RING finger proteins play crucial roles in cell-cycle progression, differentiation, development, oncogenesis, signal transduction, apoptosis and are also essential components of the cellular ubiquitin-proteasome system, which removes misfolded proteins (Borden and Freemont 1996).

Interactors BAX, BAG1 and BAD were decreased in expression at similar time points. BAG and BAD induce apoptosis via BCL2/X, BAG is also associated with BCL2, but prevents apoptosis (Takayama et al. 1995; Willis et al. 2003; Yang et al. 1995) and is therefore potentially promoting apoptosis in this study since it’s own expression is decreased.

Interactors UTRN and BCAP31 were also observed to change in their expression. CASP8 activates the BCAP31 fragment which recruits UTRN causing the scission of mitochondria (Breckenridge et al. 2003; Chandra et al. 2004). UTRN gene expression was increased and BCAP31 decreased, although in different samples to UTRN.

Interactors SIRT1, SIRT2 and FOXO3A were all present in the Apoptosis Differentials List, (Brunet et al. 2004), demonstrated that in mammalian cells, SIRT1 has a dual effect on FOXO3 function: SIRT1 increased FOXO3's ability to induce cell cycle arrest and resistance to oxidative stress but inhibited FOXO3's ability to induce cell death (Brunet et al. 2004). In this study, SIRT1 is increased and FOXO3A decreased, appearing to support this theory. SIRT2 expression was also decreased although the significance of this observation is unknown.

APP and APLP2 were observed increased in expression, APP is directly and efficiently cleaved by caspases (mostly CASP3) during apoptosis resulting in elevated amyloid beta formation. (Gervais et al. 1999). APP produces ABETA and AID, AID lowers cellular threshold to apoptosis and represses NOTCH dependent gene expression, cleaved APLP2 acts in a similar way to AID through CASP3 and 9 (Cowan et al. 2001; Scheinfeld et al. 2002). Interactors JUN, JUNB, JUND and FOS are all present and can form heterodimers and AP1 complexes, they can however act independently in apoptosis as appears to be occurring in this study (Wang et al. 2000).

**REFERENCES**

Ahmed, M.M. 2004. Regulation of radiation-induced apoptosis by early growth response-1 genes in solid tumors. *Current cancer drug targets* **1:** 43-52.

Akiyama, T., P. Bouillet, T. Miyazaki, Y. Kadono, H. Chikuda, U.I. Chung, A. Fukuda, A. Hikita, H. Seto, T. Okada, T. Inaba, A. Sanjay, R. Baron, H. Kawaguchi, H. Oda, K. Nakamura, A. Strasser, and S. Tanaka. 2003. Regulation of osteoclast apoptosis by ubiquitylation of proapoptotic BH3-only Bcl-2 family member Bim. *The EMBO Journal* **22:** 6653-6664.

Arnold, N.B., K. Ketterer, J. Kleeff, H. Friess, M.W. Buchler, and M. Korc. 2004. Thioredoxin is downstream of Smad7 in a pathway that promotes growth and suppresses cisplatin-induced apoptosis in pancreatic cancer. *Cancer research* **64:** 3599-3606.

Baek, S.J., J.S. Kim, F.R. Jackson, T.E. Eling, M.F. McEntee, and S.H. Lee. 2004. Epicatechin gallate-induced expression of NAG-1 is associated with growth inhibition and apoptosis in colon cancer cells. *Carcinogenesis* **25:** 2425-2432.

Barrett, A.J. and N.D. Rawlings. 2001. Evolutionary lines of cysteine peptidases. *Biological chemistry* **382:** 727-733.

Barton, B.E., J.G. Karras, T.F. Murphy, A. Barton, and H.F. Huang. 2004. Signal transducer and activator of transcription 3 (STAT3) in prostate cancer: Direct STAT3 inhibition induces apoptosis in prostate cancer lines. *Molecular cancer therapy* **3:** 11-20.

Baumann, R., C. Casaulta, D. Simon, S. Conus, S. Yousefi, and H.U. Simon. 2003. Macrophage migration inhibitory factor delays apoptosis in neutrophils by inhibiting the mitochondria-dependent death pathway. *FASEB* **17:** 2221-2230.

Betsholtz, C., A. Johnsson, C.-H. Heldin, B. Westermark, P. Lind, M.S. Urdea, R. Eddy, T.B. Shows, K. Philpott, A.L. Mellor, T.J. Knott, and J. Scott. 1986. cDNA sequence and chromosomal localization of human platelet-derived growth factor A-chain and its expression in tumour cell lines. *Nature* **320:** 695-699.

Bollain-y-Goytia, J.J., E. Avalos-Diaz, and R. Herrar-Esparza. 2000. Fas ligand and Bax gene transcription contributes to Ro60 ribonucleoprotein redistribution in UV-A irradiated human keratinocytes. *Joint bone and spine* **67:** 283-289.

Brazas, R. and D. Ganem. 1996. A cellular homolog of hepatitis delta antigen: implications for viral replication and evolution. *Science* **274:** 90-94.

Brekenridge, D.G., M. Stojanovic, R.C. Marcellus, and G.C. Shore. 2003. Caspase cleavage product of BAP31 induces mitochondrial fission through endoplasmic reticulum calcium signals, enhancing cytochrome c release to the cytosol. *Journal of cell biology* **160:** 1115-1127.

Bruick, R.K. 2000. Expression of the gene encoding the proapoptotic Nip3 protein is induced by hypoxia. *Proceedings of the National Academy of Science* **97:** 9082-9087.

Brunet, A., L.B. Sweeney, J.F. Sturgill, K.F. Chua, P.L. Greer, Y. Lin, H. Tran, S.E. Ross, R. Mostolavsky, H.Y. Cohen, L.S. Hu, H.-L. Cheng, M.P. Jedrychowski, S.P. Gygi, D.A. Sinclair, F.W. Alt, and M.E. Greenberg. 2004. Stress-dependent regulation of FOXO transcription factors by the SIRT1 deacetylase. *Science* **303:** 2011-2015.

Callahan, J., J. Aster, J. Sklar, E. Kieff, and E.S. Robertson. 2000. Intracellular forms of human NOTCH1 interact at distinctly different levels with RBP-jkappa in human B and T cells. *Leukemia research* **14:** 84-92.

Cates, C.A., R.L. Michael, K.R. Stayrook, K.A. Harvey, Y.D. Burke, S.K. Randall, P.L. Crowell, and D.N. Crowell. 1996. Prenylation of oncogenic human PTP(CAAX) protein tyrosine phosphatases. *Cancer letters* **110:** 49-55.

Cavenagh, M.M., M. Breiner, A. Schurmann, A.G. Rosenwald, T. Terui, C. Zhang, P.A. Randazzo, M. Adams, H.G. Joost, and R.A. Kahn. 1994. ADP-ribosylation factor (ARF)-like 3, a new member of the ARF family of GTP-binding proteins cloned from human and rat tissues. *Journal of biological chemistry.* **269:** 18937-18942.

Chandra, D., G. Choy, X. Deng, B. Bhatia, P. Daniel, and D.C. Tang. 2004. Association of active caspase 8 with the mitochondrial membrane during apoptosis: potential roles in cleaving BAP31 and caspase 3 and mediating mitochondrion-endoplasmic reticulum cross talk in etoposide-induced cell death1. *Molecular cell biology* **24:** 6592-6607.

Chen, F., M. Kamradt, M. Mulcahy, Y. Byun, H. Xu, M.J. McKay, and V.L. Cryns. 2002. Caspase proteolysis of the cohesin component RAD21 promotes apoptosis. *Journal of biological chemistry* **277:** 16775-16781.

Cole, D.S. and P.B. Morgan. 2003. Beyond lysis: how complement influences cell fate. *Clinical Science* **104:** 455-466.

Dallol, A., D. Morton, E.R. Maher, and F. Latif. 2003. SLIT2 axon guidance molecule is frequently inactivated in colorectal cancer and suppresses growth of colorectal carcinoma cells. *Cancer research* **63:** 1054-1058.

Deiss, L., E. Feinstein, H. Berissi, O. Cohen, and A. Kimchi. 1995. Identification of a novel serine/threonine kinase and a novel 15-kD protein as potential mediators of the gamma interferon-induced cell death. *Genes and development* **9:** 15-30.

Donahoe, P.K., T. Clarke, J. Texeira, S. Maheswaran, and D.T. MacLaughlin. 2003. Enhanced purification and production of Mullerian inhibiting substance for therapeutic applications. *Molecular and cellular endocrinology* **211:** 37-42.

Drivdahl, R., K.H. Haugk, C.C. Sprenger, P.S. Nelson, M.K. Tennant, and S.R. Plymate. 2004. Suppression of growth and tumorigenicity in the prostate tumor cell line M12 by overexpression of the transcription factor SOX9. *Oncogene* **23:** 4584-4593.

Du, C., M. Fang, Y. Li, L. Li, and X. Wang. 2000. Smac, a mitochondrial protein that promotes cytochrome c-dependent caspase activation by eliminating IAP inhibition. *Cell* **102:** 33-42.

Eberle, F., P. Dubreuil, M.-G. Mattei, E. Devilard, and M. Lopez. 1995. The human PRR2 gene, related to the human poliovirus receptor gene (PVR), is the true homolog of the murine Mph gene. *Gene* **159:** 267-272.

Evans, P.C., E.R. Taylor, J. Coadwell, K. Heyninck, R. Beyaert, and P.J. Kilshaw. 617-623. Isolation and characterization of two novel A20-like proteins. *Biochemistry* **357:** 617-623.

Fischer, W.H. and D. Schubert. 1996. Characterization of a novel platelet-derived growth factor-associated protein. *Journal of neurochemistry* **66:** 2213-2216.

Fishelson, Z., N. Donin, S. Zell, S. Schultz, and M. Kirschfink. 2003. Obstacles to cancer immunotherapy: expression of membrane complement regulatory proteins (mCRPs) in tumors. *Molecular immunology* **40:** 109-123.

Fitter, S., T. T.J., M.C. Berndt, and L.K. Ashman. 1995. Molecular cloning of cDNA encoding a novel platelet-endothelial cell tetra-span antigen, PETA-3. *Blood* **86:** 1348-1355.

Fortin, A., J.G. MacLaurin, N. Arbour, S. Cregan, P., N. Kushwaha, S.M. Callaghan, D.S. Park, P.R. Albert, and R.S. Slack. 2004. The proapoptotic gene SIVA is a direct transcriptional target for tumor suppressors p53 and E2F1. *Journal of biological chemistry* **279:** 28706-28714.

Fu, M., F.M. Wang, X. Zhang, and R.G. Pestell. 2004. Acetylation of nuclear receptors in cellular growth and apoptosis. *Biochemical pharmacology* **68:** 1199-1208.

Gervais, F.G., D. Xu, G.S. Robertson, J.P. Vaillancourt, Y. Zhu, J. Huang, A. LeBlanc, D. Smith, M. Rigby, M.S. Shearman, E.E. Clarke, H. Zheng, L.H.T. Van Der Ploeg, S.C. Ruffolo, N.A. Thornberry, S. Xanthoudakis, R.J. Zamboni, S. Roy, and D.W. Nicholson. 1999. Involvement of caspases in proteolytic cleavage of Alzheimer's amyloid-beta precursor protein and amyloidogenic A-beta peptide formation. *Cell* **97:** 395-406.

Ginsberg, D. 2004. E2F3-a novel repressor of the ARF/p53 pathway. *Development of the Cell* **2:** 261-272.

Golay, J., L. Zaffaroni, T. Vaccari, M. Lazzari, G.M. Borleri, S. Bernasconi, F. Tedesco, A. Rambaldi, and M. Introna. 2000. Biologic response of B lymphoma cells to anti-CD20 monoclonal antibody rituximab in vitro: CD55 and CD59 regulate complement-mediated cell lysis. *Blood* **95:** 3900-3908.

Harman, M.G., D. Lu, M.L. Kim, G.J. Kociba, T. Shukri, J. Buteau, X. Wang, W.L. Frankel, D. Guttridge, M. Prentki, S.T. Grey, D. Ron, and T. Hai. 2004. Role for activating transcription factor 3 in stress-induced beta-cell apoptosis. *Molecular cell biology* **24:** 5721-5732.

Herin, H. and M. Sheng. 2002. Direct interaction of Frizzled -1, -2, -4, and -7 with PDZ domains of PSD-95. *FEBS Letters* **19:** 185-189.

Hitchens, M.R. and P.D. Robbins. 2003. The role of the transcription factor DP in apoptosis. *Apoptosis* **8:** 461-468.

Hoffmann, T.G., A. Moller, H. Sirma, H. Zentgraf, Y. Taya, W. Droge, H. Will, and M.L. Schmitz. 2002. Regulation of p53 activity by its interaction with homeodomain-interacting protein kinase-2. *Nature cell biology* **4:** 1-10.

Hoon, K.D., C.S. Jeon, S. Kook, W. Kim, and W. Keun Song. 2003. Phosphorylation-dependent cleavage of p130cas in apoptotic rat-1 cells. *Biochemical and biophysical research communications* **300:** 141-148.

Hu, R.-J., M.P. Lee, L.A. Johnson, and A.P. Feinberg. 1996. A novel human homologue of yeast nucleosome assembly protein, 65 kb centromeric to the p57KIP2 gene, is biallelically expressed in fetal and adult tissues. *Human molecular genetics* **5:** 1743-1748.

Hubmann, R., J.D. Schwarzmeier, M. Shehata, M. Hilgarth, M. Duechler, M. Dettke, and R. Berger. 2002. Notch2 is involved in the overexpression of CD23 in B-cell chronic lymphocytic leukemia. *Blood* **99:** 3742-3747.

Huggon, I.C., A. Davies, C. Gove, G. Moscoso, C. Moniz, Y. Foss, F. Farzaneh, and P. Towner. 1997. Molecular cloning of human GATA-6 DNA binding protein: high levels of expression in heart and gut. *Biochim. biophy. acta* **1353:** 98-102.

Ihrie, R.A., E. Reczek, J.S. Horner, L. Khachatrian, J. Sage, T. Jacks, and L.D. Attardi. 2003. Perp is a mediator of p53-dependent apoptosis in diverse cell types. *Current Biology* **13:** 1985-1990.

Inizawa, J., T. Ariyama, T. Abe, T. Druck, M. Ohta, K. Huebner, J. Yanagisawa, J.C. Reed, and T. Sato. 1996. PTPN13, a Fas-associated protein tyrosine phosphatase, is located on the long arm of chromosome 4 at band q21.3. *Genomics* **31:** 240-242.

Irion, U. and M. Leptin. 1999. Developmental and cell biological functions of the Drosophila DEAD-box protein Abstrakt. *Current Biology* **9:** 1373-1380.

Ishiguro, H., T. Tsunoda, T. Tanaka, F. Y., Y. Nakamura, and Y. Furukawa. 2001. Identification of AXUD1, a novel gene induced by AXIN1 and its reduced expression in human carcinomas of the lung, liver, colon and kidney. *Oncogene* **20:** 5062-5066.

Jean, J.C., S.M. Oakes, and M. Joyce-Brady. 1999. The Bax inhibitor-1 gene is differentially regulated in adult testis and developing lung by two alternative TATA-less promoters. *Genomics* **57:** 201-208.

Jia, S.H., Y. Li, J. Parodo, A. Kapus, L. Fan, O.D. Rotstein, and J.C. Marshall. 2004. Pre-B cell colony-enhancing factor inhibits neutrophil apoptosis in experimental inflammation and clinical sepsis. *Journal of clinical investigation* **113:** 1318-1327.

Jullien-Flores, V., O. Dorseuil, R. Romero, F. Letourneur, S. Saragosti, R. Berger, A. Tavitian, G. Gacon, and J.H. Camonis. 1995. Bridging Ral GTPase to Rho pathways: RLIP76: a Ral effector with CDC42/Rac GTPase-activating protein activity. *Journal of biological chemistry* **270:** 22473-22477.

Kanekura, T., T. Miyauchi, A. Kuwano, Y. Matsuda, T. Muramatsu, and T. Kajii. 1991. Basigin, a new member of the immunoglobulin superfamily: genes in different mammalian species, glycosylation changes in the molecule from adult organs and possible variation in the N-terminal sequences. *Cell structure and function* **16:** 23-30.

Kawai, T., M. Matsumoto, K. Takeda, H. Sanjo, and S. Akira. 1998. ZIP kinase, a novel serine/threonin kinase which mediates apoptosis. *Molecular cell biology* **18:** 1642-1651.

Keller, E.T. 2004. Metastasis suppressor genes: a role for raf kinase inhibitor protein (RKIP). *Anticancer drugs* **15:** 663-669.

Kim, M.L., M. Sgagia, X. Deng, Y.J. Jung, T. Rikiyama, K. Lee, M. Ouellete, and K. Cowan. 2004. Apoptosis induced by adenovirus-mediated p14ARF expression in U2OS osteosarcoma cells is associated with increased Fas expression. *Biochemical and biophysical research communications* **320:** 138-144.

Kim, T.Y., K.H. Lee, S. Chang, C. Chung, H.W. Lee, J. Yim, and T.K. Kim. 2003. Oncogenic potential of a dominant negative mutant of interferon regulatory factor 3. *Journal of biological chemistry* **278:** 15272-15278.

Kirikoshi, H. and M. Katoh. 2002. Expression of WNT7A in human normal tissues and cancer, and regulation of WNT7A and WNT7B in human cancer. *International journal of oncology* **21:** 895-900.

Lee, S.J., H.J. Yoo, Y.S. Bae, H.J. Kim, and S.T. Lee. 2003. TIMP-1 ihibits apoptosis in breast carcinoma cells via a pathway involving pertussis toxin-sensitive G protein and c-Src. *Biochemistry and biophysics research communication* **312:** 1196-1201.

Leverrier, Y. and A.J. Ridley. 2001. Requirement for Rho GTPases and PI 3-kinases during apoptotic cell phagocytosis by macrophages. *Current Biology* **11:** 195-199.

Li, F., G. Ambrosini, E.Y. Chu, J. Plescia, S. Tognin, P.C. Marchisio, and D.C. Altieri. 1998. Control of apoptosis and mitotic spindle checkpoint by survivin. *Nature* **396**.

Lin, Y., W. Ma, and S. Benchimol. 2000. Pidd, a new death-domain-containing protein, is induced by p53 and promotes apoptosis. *Nature Genetics* **26:** 122-127.

Linenberger, M.L., J.L. Rohn, T. Deng, S. Ellis-Smith, R. Ingber, and J. Overbaugh. 1999. Lymphokines modulate the growth and survival of thymic tumor cells containing a novel feline leukemia virus/Notch2 variant. *70***:** 223-243.

Liu, L.X., H.C. Jiang, Z.H. Liu, A.L. Zhu, J. Zhou, W.H. Zhang, X.Q. Wang, and M. Wu. 2003. Gene expression profiles of hepatoma cell line BEL-7042. *Hepatogastroenterology* **50:** 1496-1504.

Liu, X., H. Zou, C. Slaughter, and X. Wang. 1997. DFF, a heterodimeric protein that functions downstream of caspase-3 to trigger DNA fragmentation during apoptosis. *Cell* **89**.

Luo, J., A.Y. Nikolaev, S. Imai, D. Chen, F. Su, A. Shiloh, L. Guarente, and W. Gu. 2001. Negative control of p53 by Sir2-alpha promotes cell survival under stress. *Cell* **107:** 137-148.

Ma, Y.X., S.Z. Zhang, Y.P. Hou, X.L. Huang, Q.Q. Wu, and Y. Sun. 2003. Identification of a novel human zince finger protein gene ZNF313. *Acta Biochimical Biophysics Singapre* **35:** 230-237.

Mancini, F., F. Gentiletti, M. D'Angelo, S. Giglio, S. Nanni, C. D'Angelo, A. Farsetti, G. Citro, A. Saachi, A. Pontecorvi, and F. Moretti. 2004. MDM4 (MDMX) overexpression enhances stabilization of stress-induced p53 and promotes apoptosis. *Journal of biological chemistry* **279:** 8169-8180.

Manna, P.P. and W.A. Frazer. 2004. CD47 mediates killing of breast tumor cells via Gi-dependent inhibition of protein kinase A. *Cancer research* **64:** 1026-1036.

Mathas, S., M. Hinz, I. Anagnostopoulos, D. Krappmann, A. Lietz, F. Jundt, K. Bommert, F. Mechta-Grigoriou, H. Stein, B. Dorken, and C. Scheidereit. 2002. Aberrantly expressed c-Jun and JunB are a hallmark of Hodgkin lymphoma cells, stimulate proliferation and synergize with NF-kappa-B. *EMBO* **21:** 4104-4113.

Matsuura, H., H. Nishitoh, K. Takeda, A. Matsuzawa, T. Amagasa, M. Ito, K. Yoshioka, and H. Ichijo. 2002. Phosphorylation-dependent scaffolding role of JSAP1/JIP3 in the ASK1-JNK signaling pathway. A new mode of regulation of the MAP kinase cascade. *Journal of biological chemistry* **277:** 40703-40709.

Mikosz, C.A., D.R. Brickley, M.S. Sharkey, T.W. Moran, and S.D. Conzen. 2001. Glucocorticoid receptor-mediated protection from apoptosis is associated with induction of the serine/threonine survival kinase gene, sgk-1. *Journal of biological chemistry* **276:** 16649-16654.

Monaco, E.A.r. and M.L. Vallano. 2003. Cyclin-dependent kinase inhibitors: cancer killers to neuronal guardians. *Current medicinal chemistry* **10:** 367-379.

Moynihan, T.P., H.C. Ardley, U. Nuber, S.A. Rose, P.F. Jones, A.F. Markham, M. Scheffner, and P.A. Robinson. 1999. The ubiquitin-conjugating enzymes UbcH7 adn UbcH8 interact with RING finger/IBR motif-containing domains of HHARI and H7-AP1. *Journal of biological chemistry* **274:** 30963-30968.

Murayama, Y., J. Miyagawa, K. Oritani, H. Yoshida, K. Yamamoto, O. Kishida, T. Miyazaki, S. Tsutsui, T. Kiyohara, Y. Miyazaki, S. Higashiyama, Y. Matsuzawa, and Y. Shinomura. 2004. CD9-mediated activation of the p46 Shc isoform leads to apoptosis in cancer cells. *Journal of cell science* **117:** 3379-3388.

Muromoto, R., K. Sugiyama, A. Takachi, S. Imoto, N. Sato, T. Yamamoto, K. Oritani, K. Shimoda, and T. Matsuda. 2004. Physical and functional interactions between Daxx and DNA methyltransferase 1-associated protein, DMAP1. *Journal of immunology* **172:** 2985-2993.

Muromoto, R., T. Yamamoto, T. Yumioka, Y. Sekine, K. Sugiyama, K. Shimoda, K. Oritani, and T. Matsuda. 2003. Daxx enhances Fas-mediated apoptosis in a murine pro-B cell line, BAF3. *FEBS Letters* **540:** 223-228.

Nagase, T., R. Kikuno, K. Ishikawa, M. Hirosawa, and O. Ohara. 2000. Prediction of the coding sequences of unidentified human genes. XVI. The complete sequences of 150 new cDNA clones from brain which code for large proteins in vitro. *DNA research* **7:** 65-73.

Nagasko, T., T. Sugiyama, T. Mizushima, Y. Miura, M. Kato, and M. Asaka. 2003. Up-regulated Smad5 mediates apoptosis of gastric epithelial cells induced by Helicobacter pylori infection. *Journal of biological chemistry* **278:** 4821-4825.

Nakamura, H., H. Tsuiki, Y. Honda, J. Sasaki, N. Masuko, K. Akagi, and H. Saya. 1997. Identification of a gene that inhibits p53-induced apoptosis. *EMBL/GenBank/DDBJ databases.* **FEB-1997**.

Nakashima, T., T. Sekiguchi, A. Kuraoka, K. Fukushima, Y. Shibata, S. Komiyama, and T. Nishimoto. 1993. Molecular cloning of a human cDNA encoding a novel protein, DAD1, whoe defect causes apoptotic cell death in hamster BHK21 cells. *Molecular cell biology* **13:** 6367-6374.

Nguyen, M.D., W.E. Mushynski, and J.P. Julien. 2002. Cycling at the interface between neurodevelopment and neurodegeneration. *Cell death differentiation* **9:** 1294-1306.

Ohlssen, M., R. Jonsson, and K.A. Brokstad. 2002. Subcellular redistribution and surface exposure of the Ro52, Ro60 and La48 autoantigens during apoptosis in human ductal epithelial cells: a possible mechanism in the pathogenesis of Sjogren's sydrome. *Scandinavian journal of immunology* **56:** 456-469.

Okumura, H., N. Nagay, T. Itoh, I. Okano, J. Hino, K. Mori, Y. Tsukamoto, H. Ishibashi-Ueda, S. Miwa, K. Tambara, S. Toyokuni, C. Yutani, and K. Kangawa. 2004. Adrenomedullin attenuates myocardial ischemia/reperfusion injury through the phosphoatidylinositol 3-kinase/Akt-dependent pathway. *Circulation* **109:** 242-248.

Opitz, J.M. 1987. G syndrome (hypertelorism with esophageal abnormality and hypospadias, or hypospadias-dysphagia, or "Opitz-Frias" or "Opitz-G" syndrome)--perspective in 1987 and bibliography. *American Journal of Medical Genetics* **28:** 275-285.

Orth, K., L.E. Palmer, Z.Q. Bao, S. Stewart, A.E. Rudolph, J.B. Bliska, and J.E. Dixon. 1999. Inhibition of the mitogen-activated protein kinase kinase superfamily by a Yersinia effector. *Science* **285:** 1920-1923.

Padilla, P.I., M.J. Chang, G. Pacheco-Rodriguez, R. Adamik, J. Moss, and M. Vaughan. 2003. Interaction of FK506-binding protein 13 with brefeldin A-inhibited guanine nucleotide-exchange protein 1 (BIG1): effects of FK506. *Proceedings of the National Academy of Science* **100:** 2322-2327.

Pati, D., N. Zhang, and S.E. Plon. 2002. Linking sister chromatid cohesion and apoptosis: role of Rad21. *Molecular cell biology* **22:** 8267-8277.

Pleschka, S., T. Wolff, C. Ehrhardt, G. Hobom, O. Planz, U.R. Rapp, and S. Ludwig. 2001. Influenza virus propagation is impaired by inhibition of the Raf/MED/ERK signalling cascade. *Nature cell biology* **3:** 301-305.

Polek, T.C., M. Talpaz, B.G. Darnay, and T. Spivak-Kroizman. 2003. TWEAK mediates signal transduction and differentiation of RAW264-7 cells in the absence of Fn14/TweakR. Evidence for a second TWEAK receptor. *Journal of biological chemistry* **278:** 32317-32323.

Pulido, R., C. Serra-Pages, M. Tang, and M. Streuli. 1995. The LAR/PTP delta/PTP sigma subfamily of transmembrane protein-tyrosine-phosphatases: multiple human LAR, PTP delta, and PTP sigma isoforms are expressed in a tissue-specific manner and associate with the LAR-interacting protein LIP.1. *Proceedings of the National Academy of Science* **92:** 11686-11690.

Rajpal, A., Y.A. Cho, B. Yelent, P.H. Koza-Taylor, D. Li, E. Chen, M. Whang, C. Kang, T.G. Turi, and A. Winoto. 2003. Transcriptional activation of known and novel apoptotic pathways by Nur77 orphan steroid receptor. *EMBO* **15;22:** 6526-6536.

Rao, J. and N. Li. 2004. Microfilament actin remodeling as a potential target for cancer drug development. *Current cancer drug targets* **4:** 345-354.

Regnier, C.H., R. Masson, V. Kedinger, J. Textoris, I. Stoll, M.-P. Chenard, A. Dierich, C. Tomasetto, and M.-C. Rio. 2002. Impaired neural tube closure, axial skeleton malformations, and tracheal ring disruption in TRAF4-deficient mice. *Proceedings of the National Academy of Science* **99:** 5585-5590.

Reigstad, L.J., H.M. Sande, O. Fluge, O. Bruland, A. Muga, J.E. Varhaug, A. Martinez, and J.R. Lillehaug. 2003. Platelet-derived growth factor (PDGF)-C, a PDGF family member with a vascular endothelial growth factor-like structure. *Journal of biological chemistry* **278:** 17114-17120.

Saijo, K., C. Schmedt, I. Su, H. Karasuyama, C.A. Lowell, M. Reth, T. Adachi, A. Patke, A. Santana, and A. Tarakhovsky. 2003. Essential role of Src-family protein tyrosine kinases in NF-kappa-B activation during B cell development. *Nature immunology* **4:** 274-279.

Saito, A., T.K. Watanabe, Y. Shimada, T. Fujiwara, C.A. Slaughter, G.N. DeMartino, N. Tanahashi, and K. Tanaka. 1997. cDNA cloning and functional analysis of p44.5 and p55, two regulatory subunits of the 26S proteasome. *Gene* **203:** 241-250.

Saitoh, T., M. Hirai, and M. Katoh. 2001. Molecular cloning and characterization of human Frizzled-8 gene on chromosome 10p11.2. *International oncology* **18:** 991-996.

Sentfleben, U., Y. Cao, G. Xiao, F.R. Greten, G. Krahn, G. Bonizzi, Y. Chen, Y. Hu, A. Fong, S.-C. Sun, and M. Karin. 2001. Activation by IKK-alpha of a second, evolutionarily conserved, NF-kappa-B signaling pathway. *Science* **293:** 1495-1499.

Song, G., X. Liao, L. Zhou, L. Wu, Y. Feng, and Z.C. Han. 2004. HI44a, an anti-CD44 monoclonal antibody, induces differentiation and apoptosis of human acute myeloid leukemia cells. *Leukemia research* **28:** 10889-10896.

Spain, B.H., K.S. Bowdish, A.R. Pacal, S.F. Staub, D. Koo, A.-Y.R. Chang, W. Xie, and J. Colicelli. 1996. Two human cDNAs, including a homolog of Arabidopsis FUS6(COP11), suppress G-protein- and mitogen-activated protein kinase-mediated signal transduction in yeast and mammalian cells. *Molecular cellular biology* **16:** 6698-6706.

Spengler, D., M. Villalba, A. Hoffmann, C. Pantaloni, S. Houssami, J. Bockaert, and L. Journot. 1997. Regulation of apoptosis and cell cycle arrest by Zac1, a novel zinc finger protein expressed in the pituitary gland and the brain. *EMBO* **16:** 2814-2825.

Srinivasula, S.M., R. Hegde, A. Saleh, P. Datta, E. Shiozaki, J. Chai, R.-A. Lee, P.D. Robbins, T. Fernandeds-Alnemri, Y. Shi, and E.S. Alnemri. 2001. A conserved XIAP-interaction motif in caspase-9 and Smac/DIABLO regulates caspase activity and apoptosis. *Nature* **410:** 112-116.

Su, H.P., K. Nakada-Tsukui, A.C. Tosello-Trampont, Y. Li, G. Bu, P.M. Henson, and K.S. Ravichandran. 2002. Interaction of CED-6/GULP, an adapter protein involved in engulfment of apoptotic cells with CED-1 and CD91/low density lipoprotein receptor-related protein (LRP). *Journal of biological chemistry* **277:** 1172-1179.

Sun, Y., M. Tan, H. Duan, and M. Swaroop. 2001. SAG/ROC/Rbx/Hrt, a zinc RING finger gene family: molecular cloning, biochemical properties and biological functions. *Antioxidant redox signal* **3:** 635-650.

Suomalainen, L., L. Dunkel, I. Ketola, M. Eriksson, K. Erkkila, R. Oksjoki, K. Taari, M. Heikinheimo, and V. Pentikainen. 2004. Activator protein-1 in human male germ cell apoptosis. *Molecular human reproduction* **10:** 743-753.

Suzuki, S., W.S. Argraves, R. Pytela, H. Arai, T. Krusius, M.D. Pierschbacher, and E. Ruoslahti. 1986. cDNA and amino acid sequences of the cell adhesion protein receptor recognising vitronectin reveal a transmembrane domain and homologies with other adhesion protein receptors. *Proceedings of the National Academy of Science* **83:** 8614-8618.

Tachibana, I., M. Imoto, P.A. Adjei, G.J. Gores, M. Subramaniam, T.C. Spelsberg, and R. Urrutia. 1997. Overexpression of the TGF-beta-regulated zinc finger encoding gene, TIEG, induces apoptosis in pancreatic epithelial cells. *Journal of clinical investigation* **99:** 2365-2374.

Takahashi, M., M. Takahashi, F. Shiohara, H. Takada, and H. Rikiishi. 2001. Effects of superantigen and lipopolysaccharide on induction of CD80 through apoptosis of human monocytes. *Infectious immunology* **69:** 3652-3657.

Takatani, T., K. Takahashi, Y. Uozumi, E. Shikata, Y. Yamamoto, T. Ito, T. Matsuda, S.W. Schaffer, Y. Fujio, and J. Azuma. 2004. Taurine inhibits apoptosis by preventing formation of the Apaf-1/caspase-9 apoptosome. *American Journal of Physiological Cell Physiology* **287:** C949-953.

Takayama, S., T. Sato, S. Krajewski, K. Kochel, S. Irie, J.A. Millan, and J.C. Reed. 1995. Cloning and functional analysis of BAG-1: a novel Bcl-2-binding protein with anti-cell death activity. *Cell* **80:** 279-284.

Takekawa, M. and H. Saito. 1998. A family of stress-inducible GADD45A-like proteins mediate activation of the stress-responsive MTK1/MEKK4 MAPKKK. *Cell* **95:** 521-530.

Tanaka, M., M. Schinke, H.S. Liao, N. Yamasaki, and S. Izumo. 2001. Nkx2.5 and Nkx2.6, homologs of Drosophila tinman, are required for development of the pharynx. *Molecular cell biology* **21:** 4391-4399.

Tapon, N., K.F. Harvey, D.W. Bell, D.C. Wahrer, T.A. Schiripo, D.A. Haber, and I.K. Hariharan. 2002. Salvador promotes both cell cycle exit and apoptosis in Drosophila and is mutated in human cancer cell lines. *Cell* **110:** 403-406.

Taylor, V. and U. Suter. 1996. Epithelial membrane protein-2 and epithelial membrane protein-3: two novel members of the peripheral myelin protein 22 gene family. *Gene* **175:** 115-120.

Tomita, H., M. Nazmy, K. Kajimoto, G. Yehia, C.A. Molina, and J. Sadoshima. 2001. Inducible cAMP early repressor (ICER) is a negative-feedback regulator of cardiac hypertrophy and an important mediator of cardiac myocyte apoptotis in response to beta-adrenergic receptor stimulation. *Circulatory respiration* **93:** 2003.

Tournier, C., P. Hess, D.D. Yang, J. Xu, T.K. Turner, A. Nimnual, D. Bar-Sagi, S.N. Jones, R.A. Flavell, and R.J. Davis. 2000. Requirement of JNK for stress-induced activation of the cytochrome c-mediated death pathway. *Science* **288:** 870-874.

Tung, R.M. and J. Blenis. 1997. A novel SPS1/STE20 homologue, KHS, activates Jun N-terminal kinase. *Oncogene* **14:** 653-659.

Ueki, H.-Y., S. Yeh, N. Fujimoto, and C. Chang. 1999. Isolation and charaterization of a novel human gene (HFB30) which encodes a protein with a RING finger motif. *Biochemical and biophysical Acta* **1445:** 232-236.

Veech, R.L. 2003. A humble hexose monophosphate pathway metabolite regulates short- and long-term control of lipogenesis. *Proceedings of the National Academy of Science* **100:** 5578-5580.

Vega, S., A.V. Morales, O.H. Ocana, F. Valdes, I. Fabregat, and M.A. Nieto. 2004. Snail blocks the cell cycle and confers resistance to cell death. *Genes and development* **15:** 1131-1143.

Verhagen, A.M., P.G. Ekert, M. Pakusch, J. Silke, L.M. Connolly, G.E. Reid, R.L. Moritz, R.J. Simpson, and D.L. Vaux. 2000. Identification of DIABLO, a mammalian protein that promotes apoptosis by binding to and antagonizing IAP proteins. *Cell* **102:** 43-53.

Vito, P., E. Lacana, and L. D'Adamio. 1996. Interfering with apoptosis: Ca(2+)-binding protein ALG-2 and Alzheimer's disease gene ALG-3. *Science* **271:** 521-524.

Vorotnikova, E., M. Tries, and S. Branhut. 2004. Retinoids and TIMP1 prevent radiation-induced apoptosis of capillary endothelial cells. *Radiation research* **161:** 174-184.

Walczak, H., M.A. Degli-Esposti, R.S. Johnson, P.J. Smolak, J.Y. Waugh, N. Boiani, M.S. Timour, M.J. Gerhart, K.A. Schooley, C.A. Smith, R.G. Goodwin, and C.T. Rauch. 1997. TRAIL-R2: a novel apoptosis-mediating receptor for TRAIL. *EMBO* **16:** 5386-5397.

Wang, J., R.D. Schreiber, and I.L. Campbell. 2002. STAT1 deficiency unexpectedly and markedly exacerbates the pathophysiological actions of IFN-alpha in the central nervous system. *Proceedings of the National Academy of Science* **99:** 16209-16214.

Weiner, A.M. and N. Maizels. 1999. Enhanced: A deadly double life. *Science* **284:** 63-64.

Weitzman, J.B., L. Fiette, K. Matsuo, and M. Yaniv. 2000. JunD protects cells from p53-dependent senescence and apoptosis. *Molecular cell* **6:** 1109-1119.

Wiener, Z., E.C. Ontsouka, S. Jakob, R. Torgler, A. Falus, C. Mueller, and T. Brunner. 2004. Synergistic induction of the Fas (CD95) ligand promoter by Max and NFkappaB in human non-small lung cancer cells. *Experimental cell research* **299:** 227-235.

Willis, S., C.L. Day, M.G. Hinds, and D.C. Huang. 2003. The Bcl-2-regulated apoptotic pathway. *Journal of cell science* **116:** 4065-4066.

Wilson, M. 2002. Cell signaling pathways that regulate apoptotic machinery. *Innovations in cellular dynamics* **1:** 1-10.

Winograd, J., M.P. Reilly, R. Roe, J. Lutz, E. Laughner, X. Xu, L. Hu, T. Asakura, C. vander Kolk, J.D. Strandberg, and G.L. Semenza. 1997. Perinatal lethality and multiple craniofacial malformations in MSX2 transgenic mice. *Human molecular genetics* **6:** 369-378.

Wu, Y., L. Chen, P.S. Zheng, and B.B. Yang. 2002. beta-1 integrin-mediated glioma cell adhesion and free radical-induced apoptosis are regulated by binding to a C-terminal domain of PG-M/versican. *Journal of biological chemistry* **277:** 12294-12301.

Xiang, J., J.M. Lahti, J. Grenet, J. Easton, and V.J. Kidd. 1994. Molecular cloning and expression of alternatively spliced PITSLRE protein kinase isoforms. *Journal of biological chemistry* **269**.

Xu, Y.-H. and G.A. Grabowski. 1999. Molecular cloning and characterization of a translational inhibitory protein that binds to coding sequences of human acid beta-glucosidase and other mRNAs. *Molecular genetics of metabolism* **68:** 441-454.

Yang, E., J. Zha, J. Jockel, L.H. Boise, C.B. Thompson, and S.J. Korsmeyer. 1995. Bad, a heterodimeric partner for Bcl-X(L) and Bcl-2, displaces Bax and promotes cell death. *Cell* **80:** 285-291.

Yang, X., R. Khosravi-Far, H.Y. Chang, and D. Baltimore. 1997. Daxx, a novel Fas-binding protein that activates JNK and apoptosis. *Cell* **89:** 1067-1076.

Zeng, Q., J.M. Dong, K. Guo, J. Li, H.X. Tan, V. Koh, C.J. Pallen, E. Manser, and W. Hong. 2003. PRL-3 and PRL-1 promote cell migration, invasion, and metastasis. *Cancer research* **63:** 2716-2722.

Zhang, C., Y. Xu, J. Gu, and S.F. Schlossman. 1998. A cell surface receptor defined by a mAb mediates a unique type of cell death similar to oncosis. *Proceedings of the National Academy of Science* **95:** 6290-6295.
