# Supplementary material for: Identification and characterisation of human apoptosis inducing proteins using cell-based transfection microarrays and expression analysis
Source: BMC Genomics. 2006 Jun 12;7:145. doi: 10.1186/1471-2164-7-145 (PMC1525185; doi:10.1186/1471-2164-7-145)
Supplement: Additional File 5 — Figure 3. ACO1 over-expression effect on apoptotic pathway. Figure 4. STK3 over-expression effect on apoptotic pathway. Figure 5. XBP1 over-expression effect on apoptotic pathway. Figure 6. STS over-expression effect on apoptotic pathway. Figure 7. Summary of over-expression effects in apoptotic pathways. (Figures 3, 4, 5, 6, 7) Adapted from the KEGG apoptotic pathway (light blue) and BD Biosciences apoptotic pathway (light orange). Genes coloured red potentially increase apoptosis, genes coloured green potentially decrease apoptosis dependent upon their expression (red and green genes may be increased or decreased in expression, see Table 2. White writing indicates the changed expression in the gene was only observed in ACO1. [file 1471-2164-7-145-S5.doc]

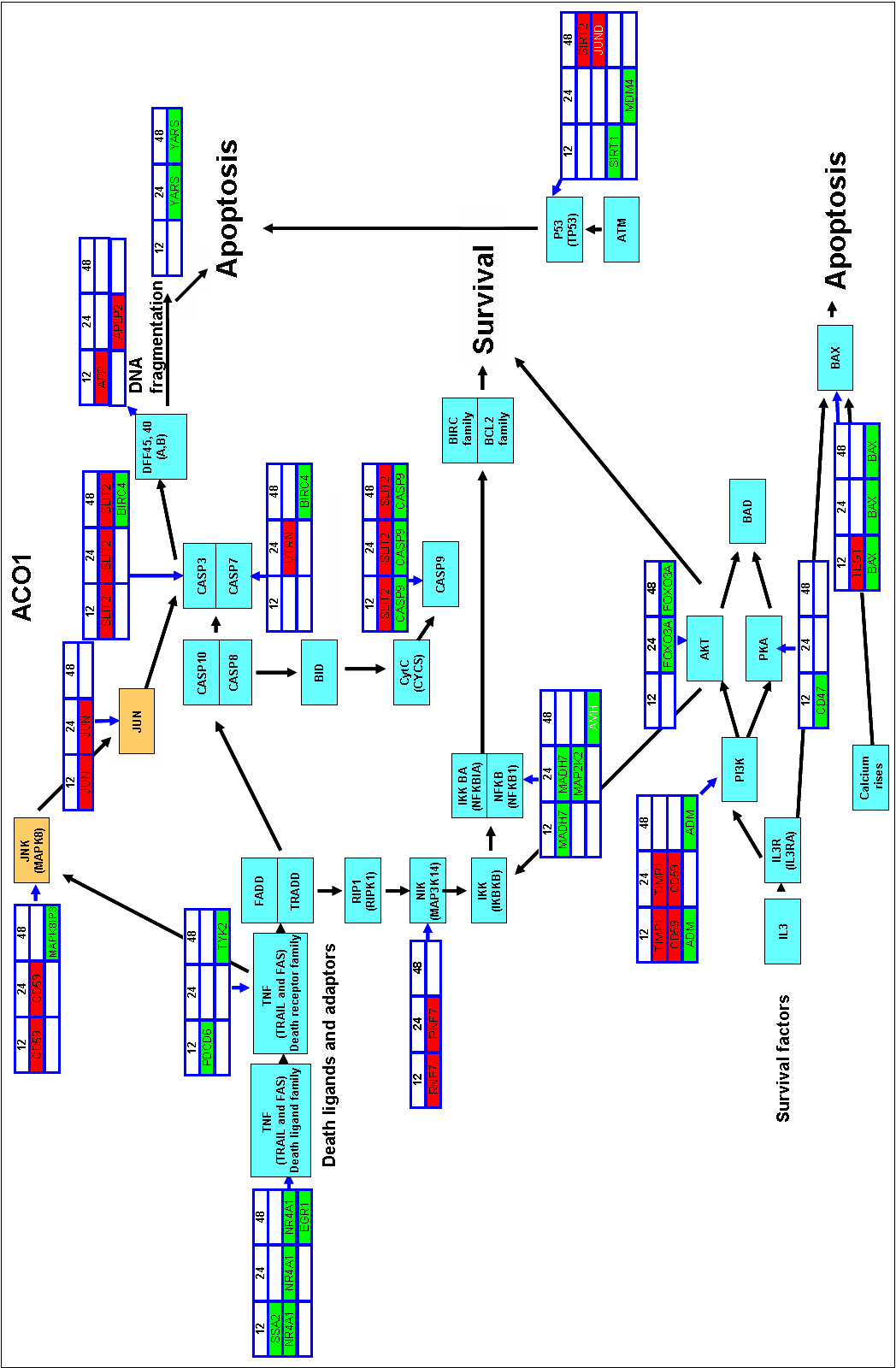


Figure 3. ACO1 over-expression effect on apoptotic pathway. Adapted from the KEGG apoptotic pathway (light blue) and BD Biosciences apoptotic pathway (light orange). Genes coloured red potentially increase apoptosis, genes coloured green potentially decrease apoptosis dependent upon their expression (red and green genes may be increased or decreased in expression, see Table 2. White writing indicates the changed expression in the gene was only observed in ACO1.


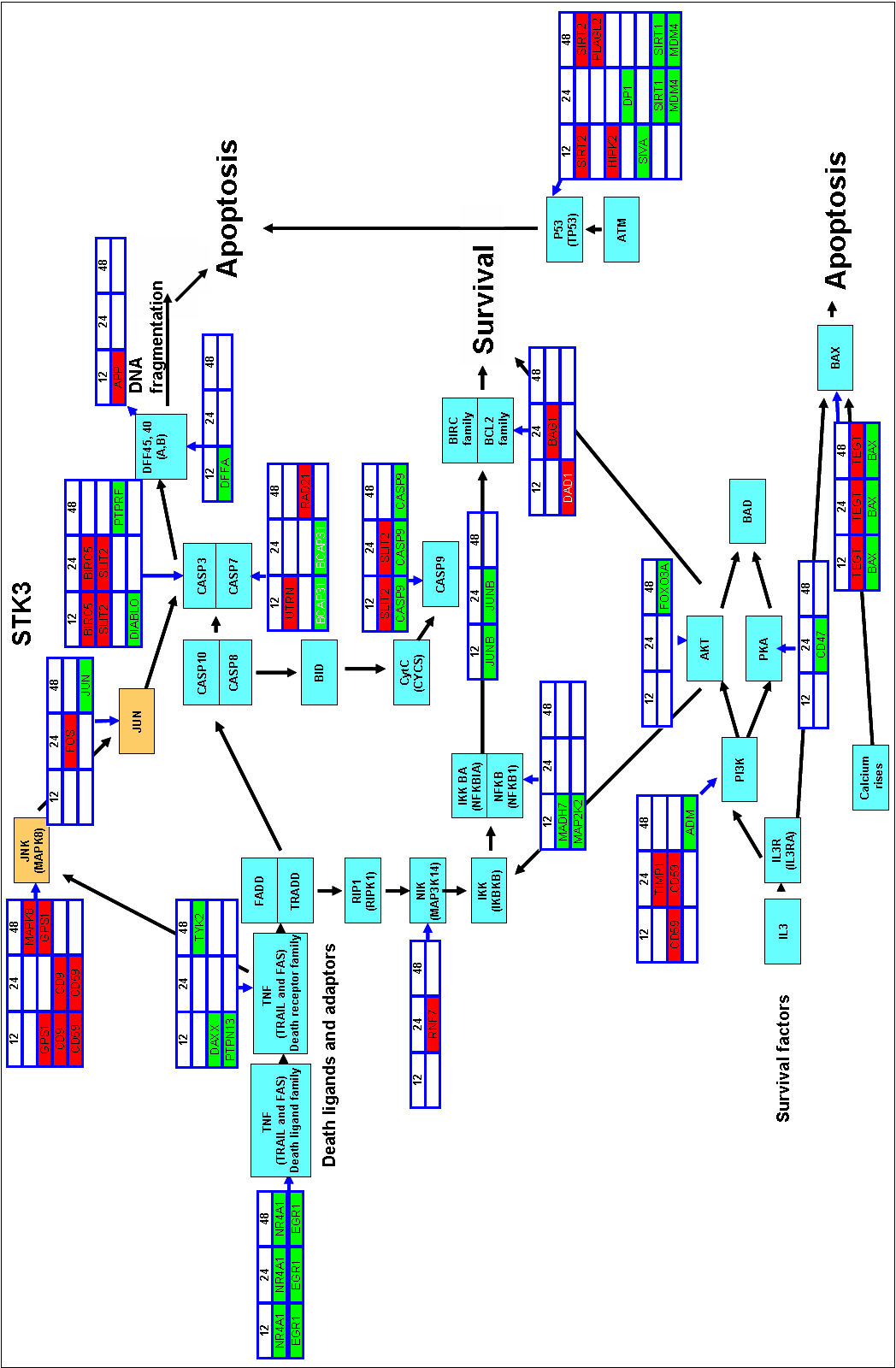


Figure 4. STK3 over-expression effect on apoptotic pathway. Adapted from the KEGG apoptotic pathway (light blue) and BD Biosciences apoptotic pathway (light orange). Genes coloured red potentially increase apoptosis, genes coloured green potentially decrease apoptosis dependent upon their expression (red and green genes may be increased or decreased in expression, see Table 2. White writing indicates the changed expression in the gene was only observed in STK3.


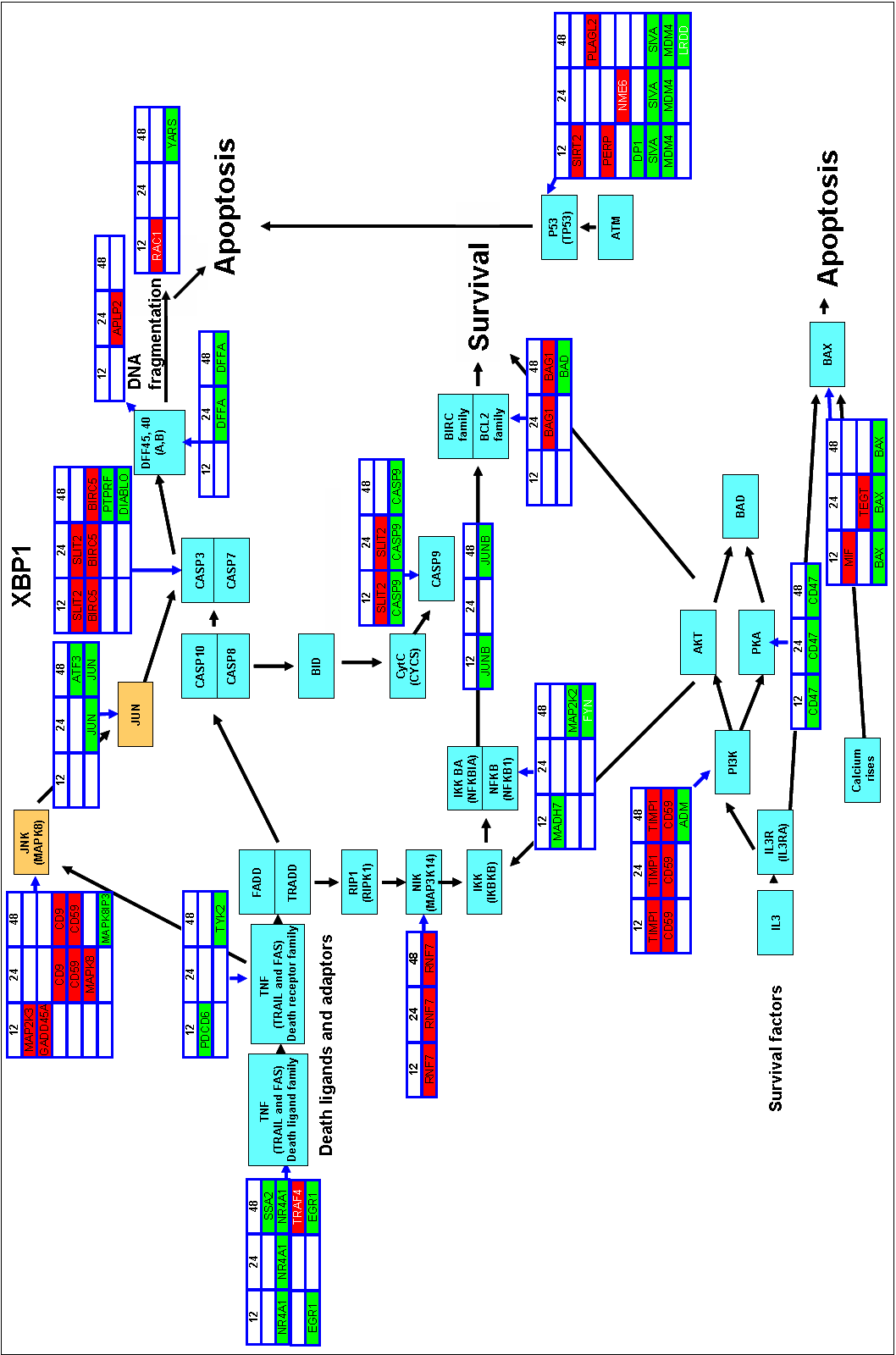


Figure 5. XBP1 over-expression effect on apoptotic pathway. Adapted from the KEGG apoptotic pathway (light blue) and BD Biosciences apoptotic pathway (light orange). Genes coloured red potentially increase apoptosis, genes coloured green potentially decrease apoptosis dependent upon their expression (red and green genes may be increased or decreased in expression, see Table 2. White writing indicates the changed expression in the gene was only observed in XBP1.


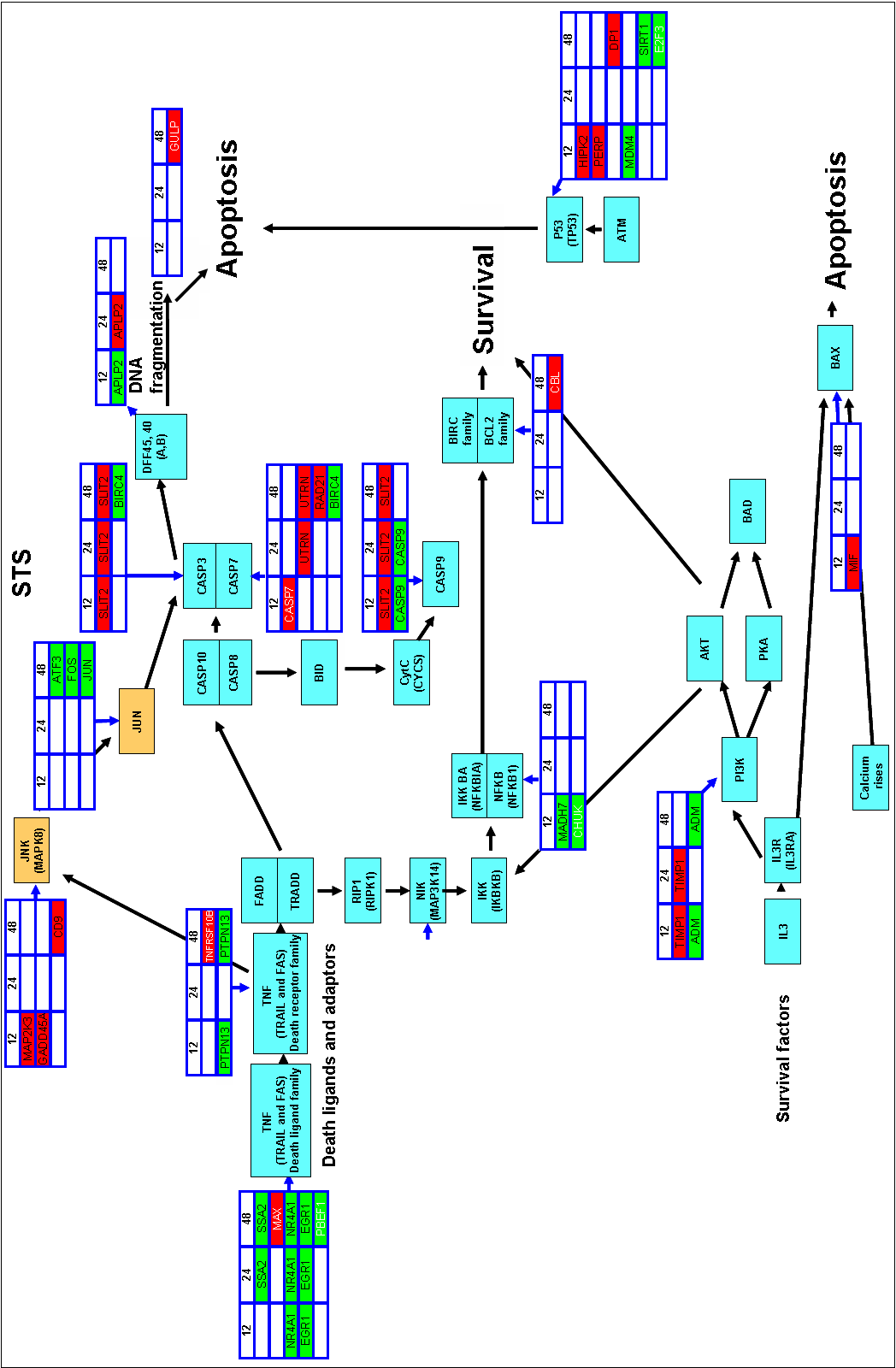


Figure 6. STS over-expression effect on apoptotic pathway. Adapted from the KEGG apoptotic pathway (light blue) and BD Biosciences apoptotic pathway (light orange). Genes coloured red potentially increase apoptosis, genes coloured green potentially decrease apoptosis dependent upon their expression (red and green genes may be increased or decreased in expression, see Table 2. White writing indicates the changed expression in the gene was only observed in STS.


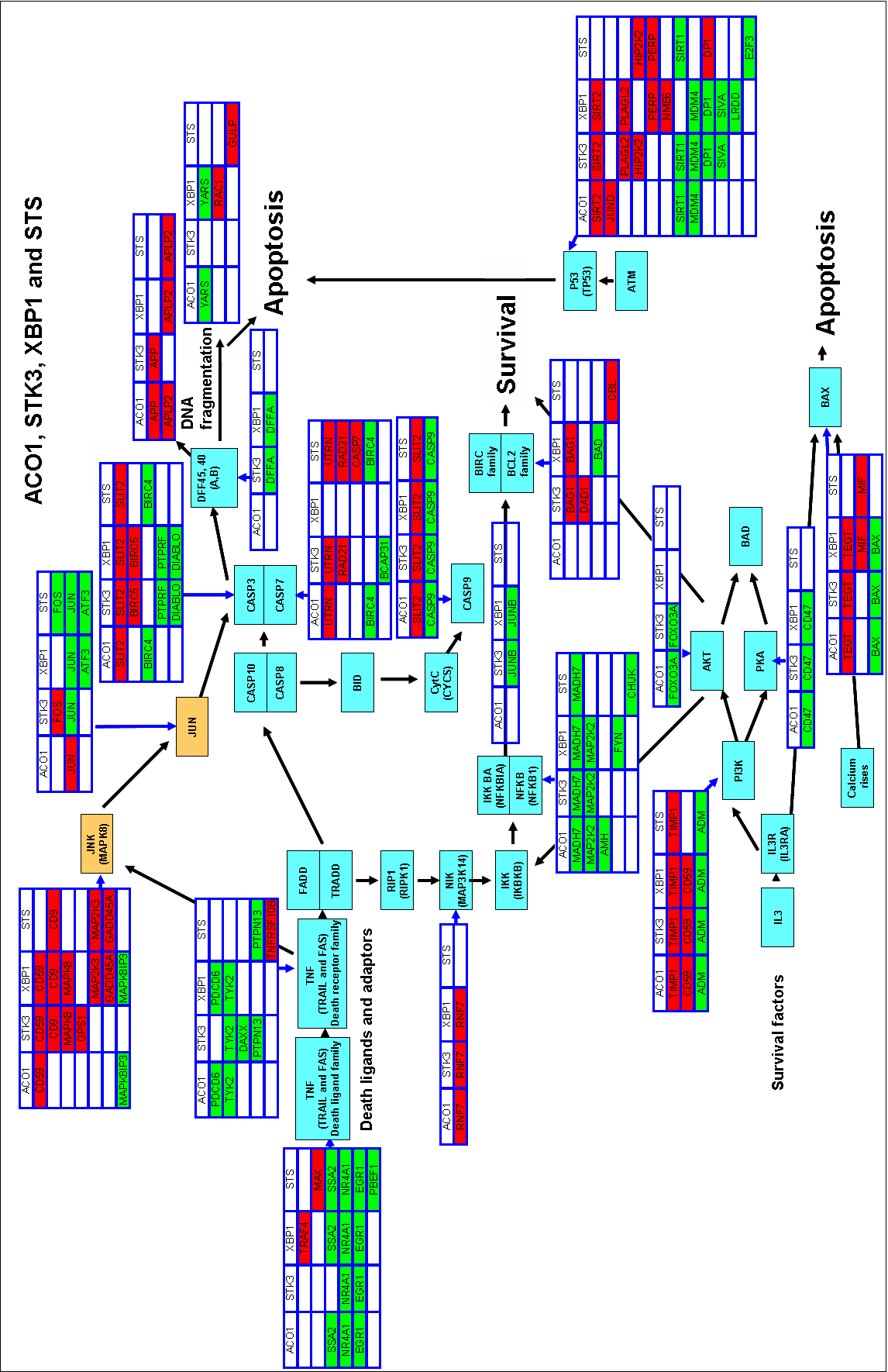


**Figure 7. Summary of over-expression effects in apoptotic pathways. Adapted from the KEGG apoptotic pathway (light blue) and BD Biosciences apoptotic pathway (light orange). Gene is shown on the pathway if present at any of the three time points in each treatment. Genes coloured red potentially increase apoptosis, genes coloured green potentially decrease apoptosis dependent upon their expression (red and green genes may be increased or decreased in expression, see Table 2).**

In all conditions, many of the genes that could potentially increase apoptosis were associated with the MAPK8/CASP3 pathway. However, in the case of XBP1 over-expression and STS treatment, the lists of apoptosis differentials also contained genes that could indicate pro-apoptotic signals were being induced in the death receptor pathways. UTRN and RAD21 were differentially expressed in cells over-expressing ACO1 and STK3 and in STS treated cells, potentially indicating apoptosis induction via CASP7. Indeed CASP7 itself was up-regulated in STS treated cells. Genes involved in DNA fragmentation and phagocytosis occurred in all of the samples, except cells over-expressing STK3. SLIT2 might potentially be increasing apoptosis via CASP3 and CASP9 in every sample. A similar number of genes over all the samples are potentially increasing and decreasing apoptosis via TP53. Differentially regulated gene transcripts appeared to be acting very similarly and NR4A1, EGR1 SLIT2, CASP9, ADM, SMAD7, JUN and TIMP1 were differentially expressed in every transfection/treatment. Two genes, JUND and AMH were observed only in cells over-expressing ACO1, three genes, DAXX, DAD1 and BCAP31 were observed only in cells over-expressing STK3, six gene transcripts, TRAF4, RAC1, NME6, BAD, FYN and LRDD were observed only in cells over-expressing XBP1 protein and eight genes MAX, PBEF1, TNFRSF10B, CASP7, GULP, CHUK, CBL and E2F3 were observed only in STS treated cells. A strong time course specific variation in the regulation of most differentially expressed genes did not appear to be evident from the data.
